# Supplementary material for: Self-similar chiral organic molecular cages
Source: Nat Commun. 2024 Jan 22;15:670. doi: 10.1038/s41467-024-44922-y (PMC10803742; doi:10.1038/s41467-024-44922-y)
Supplement: Supplementary file 1 — Supplementary Information [file 41467_2024_44922_MOESM1_ESM.pdf]

Supplementary Information

Self-Similar Chiral Organic Molecular Cages

Zhen Wang [1,2,4] ✉, Qing-Pu Zhang [1,4], Fei Guo [2,4], Hui Ma [1], Zi-Hui Liang [2], Chang-Hai Yi [2], Chun Zhang [1] ✉ & Chuan-Feng Chen [3] ✉

[1] College of Life Science and Technology, Huazhong University of Science and Technology, Wuhan, 430074, China.

[2] National Engineering Laboratory for Advanced Yarn and Fabric Formation and Clean Production, Technology Institute, Wuhan Textile University, Wuhan, Hubei 430200, China.

[3] Beijing National Laboratory for Molecular Sciences, CAS Key Laboratory of Molecular Recognition and Function, Institute of Chemistry, Chinese Academy of Sciences, Beijing, 100190, China.

[4] These authors contributed equally: Zhen Wang, Qing-Pu Zhang, Fei Guo.

Corresponding Author

Chun Zhang *e-mail:* chunzhang@hust.edu.cn;

Zhen Wang *E-mail:* wz@wtu.edu.cn;

Chuan-Feng Chen *E-mail:* cchen@iccas.ac.cn;

Contents

1. General information. S1

2. Experimental details S1

2.1 Synthesis of 1,3,5-TPB based [2+3] molecular cage (CHO-TMC). S1

2.2 Synthesis of [2[2+3]+3] higher-level molecular cage 4*P*-HTMC, 4*M*-HTMC and 4*MP*-HTMC. S3

3. ^{1}H and ^{13}C NMR spectra S7

4. 2D ^{1}H,^{1}H-COSY, ^{1}H,^{1}H-NOESY, ^{1}H,^{13}C-HSQC and ^{1}H,^{13}C-HMBC NMR spectra S11

5. ^{1}H NMR titration S18

6. MALDI-TOF mass spectrometry S19

7. X-ray single crystal diffraction structures S21

8. Computational calculation. S25

9. UV absorption spectra and Circular dichroism (CD) spectra S27

10. SEM and TEM. S29

11. PXRD S33

References S33

1. General information.

All materials obtained commercially were used without further purification. ^{1}H NMR, ^{13}C NMR, ^{1}H,^{1}H-COSY NMR, ^{1}H,^{1}H-NOESY NMR, ^{1}H,^{13}C-HSQC NMR and ^{1}H,^{13}C-HMBC NMR spectra were recorded on a DMX600 NMR. MALDI-TOF mass spectra were obtained on a BIFLEXIII mass spectrometer. CD spectra were recorded on J-810 Jasco Japan. SEM studies were conducted on JSM6510LV. TEM studies were conducted on a Tecnai G220 electron microscope. The X-ray intensity data were collected on a standard Bruker SMART-1000 CCD Area Detector System equipped with a normal-focus molybdenum-target X-ray tube (λ = 0.71073 Å) operated at 2.0 kW (50 kV, 40 mA) and a graphite monochromator. The structures were solved by using direct methods and were refined by employing full-matrix least-squares cycles on F^2 (Bruker, SHELXTL-97).

2. Experimental details

2.1 Synthesis of 1,3,5-tris(4-hydroxyphenyl)benzene (TPB) based [2+3] molecular cage (CHO-TMC).

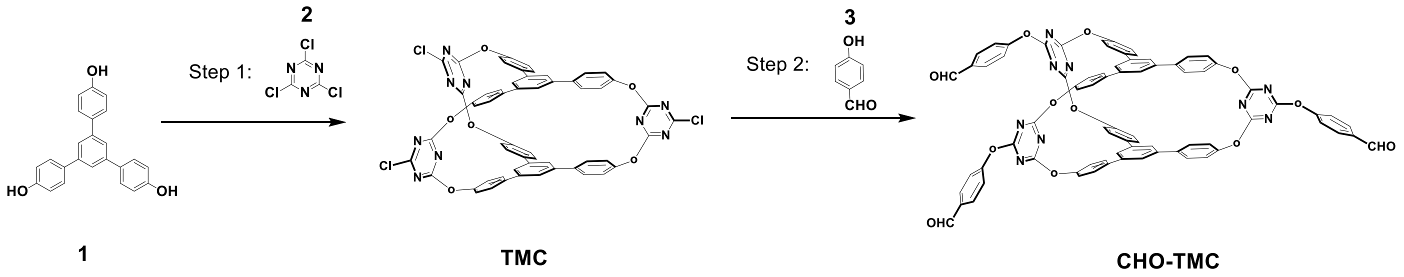

Supplementary Fig. 1. Synthesis of 1,3,5-tris(4-hydroxyphenyl)benzene TPB based [2+3] molecular cage TMC and CHO-TMC.

1,3,5-tris(4-hydroxyphenyl)benzene based [2+3] molecular cage TMC was synthesized according to the reported literature.[1] A solution of cyanuric chloride **2** (510 mg, 2.7 mmol) in 150 mL of THF, cooled with an ice bath, received a dropwise addition of a mixture containing 1,3,5-tri(4-hydroxyphenyl)benzene **1** (630 mg, 1.8 mmol) and diisopropylethylamine (840 mg, 1.14 mL, 6.48 mmol) in 40 mL of THF over 3 hours. Following

the addition, the temperature was gradually raised to 40°C, and stirring persisted for an additional 3 days. The resulting mixture was then filtered, and solvent removal was carried out using a rotary evaporator. The residue underwent chromatography on a silica gel column with petroleum ether and ethyl acetate (5:1) to yielding TMC (120 mg, 13%) with white solid. ^{1}H NMR (400 MHz, $CDCl_3$), δ (ppm) = 7.48 (s, 6H), 7.39–7.41 (d, J = 8.80 Hz, 12H); 6.94–6.96 (d, J = 8.40 Hz, 12H).

TMC (100 mg, 0.096 mmol) and 4-hydroxybenzaldehyde 3 (47 mg, 0.383 mmol) were dissolved 10 mL *N,N*-dimethylformamide (DMF), *N,N*-diisopropylethylamine (DIPEA) (67 μL, 0.383 mmol) was added, and then stirred vigorously at room temperature for 1 h. The reaction was monitored by TLC. When the raw materials were consumed, water (50 mL) was added to quench the reaction, and then filtered. The precipitate was dispersed in MeOH, ultrasonicated for 5 min and subsequently filtered, the obtained precipitate was the pure product of TPB based [2+3] molecular cage CHO-TMC (110 mg, 89%). ^{1}H NMR (600 MHz, $CDCl_3$), δ (ppm) = 10.087 (s, 3H), 8.06–8.07 (d, J = 8.40 Hz, 6H); 7.50–7.51 (d, J = 8.40 Hz, 6H), 7.48 (s, 6H), 7.38–7.40 (d, J = 8.40 Hz, 12H), 6.95–6.96 (d, J = 8.40 Hz, 12H); ^{13}C (150 MHz, $CDCl_3$): δ (ppm) = 190.83, 174.13, 173.80, 156.11, 151.57, 140.99, 138.53, 134.48, 131.59, 127.31, 123.93, 122.50., 122.30.

Crystallographic data for CHO-TMC: Mr = 1459.09, Monoclinic, Space group $C2/c$, a = 38.044(5), b = 17.566(3) Å, c = 29.995(4) Å, α = 90°, β = 123.041(4)°, γ = 90°, V = 16804(4) Å^3, Z = 8, $\rho_{calcd.}$ = 1.028 Mg/m^3, μ = 0.371 mm^{-1}, reflections collected 89115, data/restraints/parameters 14332/48/990, GOF on F^2 1.074, final R_1 = 0.0618, wR_2 = 0.1813, R indices (all data): R_1 =0.0802, wR_2 = 0.1970, largest diff. peak and hole: 0.381 and -0.314 e/Å^{-3}, CCDC - 2180996.

2.2 Synthesis of [2[2+3]+3] higher-level molecular cage 4*P*-HTMC, 4*M*-HTMC and 4*MP*-HTMC.

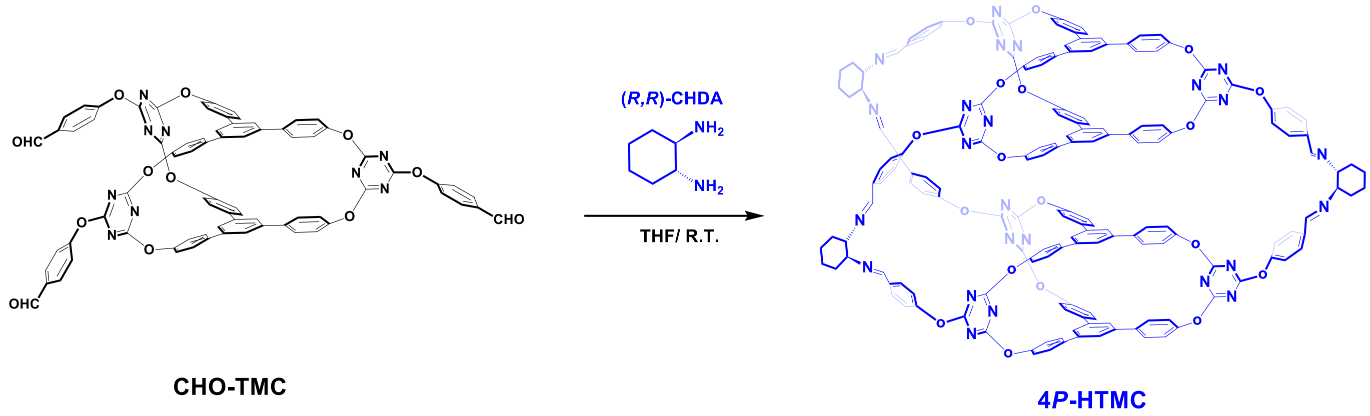

Supplementary Fig. 2. Synthesis of [2[2+3]+3] higher-level chiral molecular cage 4*P*-HTMC from 1,3,5-tris(4-hydroxyphenyl)benzene based molecular cage TMC react with (*R*,*R*)-diaminocyclohexane CHDA in tetrahydrofuran THF at room temperature.

CHO-TMC (100 mg, 0.077 mmol) and (*R*,*R*)-diaminocyclohexane (CHDA) (13.2 mg, 0.115 mmol) were dissolved in DCM (10 mL) and added to round bottom flask. Then, a catalysis amount of trifluoroacetic acid (TFA) (0.3 μL, 5% mmol) were added in. The combined mixture was stirred vigorously at room temperature for overnight. After that, the reaction was monitored by TLC with additional a small amount of (*R*,*R*)-diaminocyclohexane (CHDA). When the raw materials were consumed, the mixture was precipitated by MeOH 50 mL. The formed white precipitate was filtered and dried in vacuum to afford dry white solid molecular cage 4*P*-HTMC. (103 mg, 94%). ^{1}H NMR (600 MHz, $CDCl_3$), δ (ppm) = 8.36 (s, 6H), 7.79–7.81 (d, J = 8.40 Hz, 12H); 7.23–7.24 (d, J = 8.40 Hz, 12H), 7.19 (s, 6H), 7.15–7.16 (d, J = 8.40 Hz, 12H), 6.81–6.82 (t, 18H), 6.72–6.73 (d, J = 8.40 Hz, 12H), 6.35–6.36 (d, J = 8.40 Hz, 12H), 3.51–5.53 (m, 6H), 1.29–1.91 (m, 24H); ^{13}C (150 MHz, $CDCl_3$), δ (ppm) = 174.29, 174.09, 173.71, 159.10, 152.99, 151.64, 151.25, 141.04, 140.69, 138.59, 138.19, 134.64, 129.64, 127.23, 126.78, 124.04, 123.59, 122.37, 121.55, 121.39, 74.36, 33.21, 24.50. MALDI TOF-MS, m/z = 2835.96.

Crystallographic data for 4*P*-HTMC: Mr = 1459.09, Orthorhombic, Space group $P\ 2_1\ 2_1\ 2_1$, a =

20.2221(18), b = 29.288(3) Å, c = 40.606(4) Å, α = 90°, β = 90°, γ = 90°, V = 24049(4) Å^3, Z = 8, $\rho_{calcd.}$ = 0.981 Mg/m^3, μ = 0.256 mm^{-1}, reflections collected 116327, data/restraints/parameters 34559/166/2161, GOF on F^2 1.021, final R_1 = 0.0826, wR_2 = 0.2285, R indices (all data): R_1 = 0.1303, wR_2 = 0.2700, largest diff. peak and hole: 0.475 and -0.358 e/Å^{-3}, CCDC - 2181020.

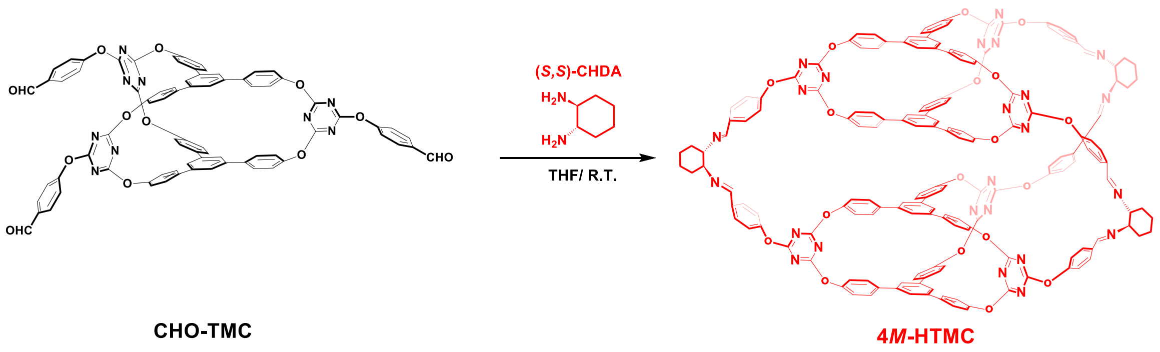

Supplementary Fig. 3. Synthesis of [2[2+3]+3] higher-level chiral molecular cage 4*M*-HTMC from 1,3,5-tris(4-hydroxyphenyl)benzene based molecular cage TMC react with (*S*,*S*)-diaminocyclohexane CHDA in tetrahydrofuran THF at room temperature.

CHO-TMC (100 mg, 0.077 mmol) and (*R*,*R*)-diaminocyclohexane (CHDA) (13.2 mg, 0.115 mmol) were dissolved in DCM (10 mL) and added to round bottom flask. Then, a catalysis amount of trifluoroacetic acid (TFA) (0.3 μL, 5% mmol) were added in. The combined mixture was stirred vigorously at room temperature for overnight. After that, the reaction was monitored by TLC with additional a small amount of (*S*,*S*)-diaminocyclohexane (CHDA). When the raw materials were consumed, the mixture was precipitated by MeOH 50 mL. The formed white precipitate was filtered and dried in vacuum to afford dry white solid molecular cage 4*M*-HTMC. (100 mg, 91%). ^{1}H NMR (600 MHz, $CDCl_3$), δ (ppm) = 8.36 (s, 6H), 7.79–7.81 (d, J = 8.40 Hz, 12H); 7.23–7.24 (d, J = 8.40 Hz, 12H), 7.19 (s, 6H), 7.15–7.16 (d, J = 8.40 Hz, 12H), 6.81–6.82 (t, 18H), 6.72–6.73 (d, J = 8.40 Hz, 12H), 6.35–6.36 (d, J = 8.40 Hz, 12H), 3.52–5.54 (m, 6H), 1.29–1.91 (m, 24H); ^{13}C (150 MHz, $CDCl_3$), δ (ppm) = 174.29, 174.10, 173.72, 159.08, 152.99, 151.65, 151.25,

141.04, 140.69, 138.59, 138.19, 134.65, 129.64, 127.23, 126.78, 124.04, 123.58, 122.37, 121.55, 121.40, 74.36, 33.21, 24.50. MALDI TOF-MS, m/z = 2835.96. MALDI TOF-MS, m/z = 2835.93.

Crystallographic data for 4*M*-HTMC: Mr = 3551.14, Orthorhombic, Space group $P\,2_1\,2_1\,2_1$, a = 19.6795(9), b = 29.1198(14) Å, c = 40.2573(18) Å, α = 90°, β = 90°, γ = 90°, V = 23070.0(18) Å^3, Z = 8, $\rho_{calcd.}$ = 0.918 Mg/m^3, μ = 1.148 mm^{-1}, reflections collected 172380, data/restraints/parameters 40238/0/2144, GOF on F^2 2.459, final R_1 = 0.2446, wR_2 = 0.5244, R indices (all data): R_1 = 0.2547, wR_2 = 0.5385, largest diff. peak and hole: 2.712 and -2.212 e/Å^{-3}, CCDC - 2181024.

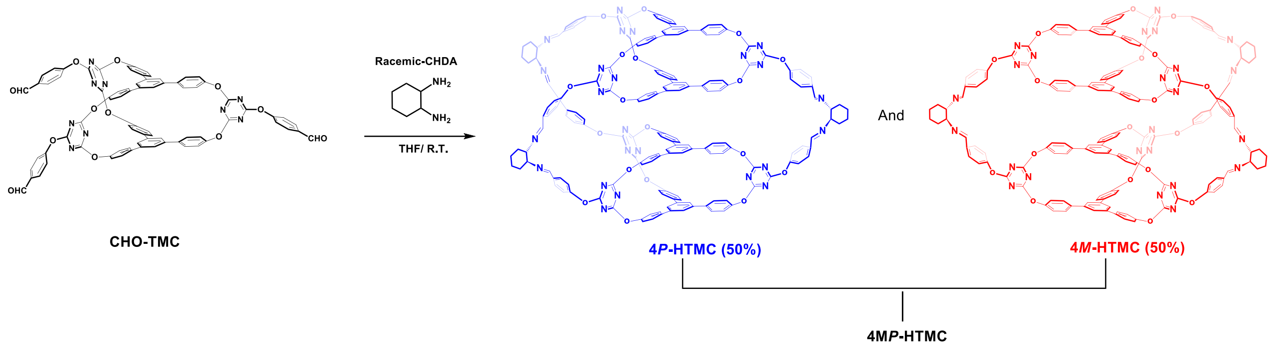

Supplementary Fig. 4. Synthesis of [2[2+3]+3] higher-level chiral molecular cage 4*MP*-HTMC from 1,3,5-tris(4-hydroxyphenyl)benzene based molecular cage TMC react with racemic diaminocyclohexane CHDA in tetrahydrofuran THF at room temperature..

CHO-TMC (100 mg, 0.077 mmol) and racemic diaminocyclohexane (CHDA) (13.2 mg, 0.115 mmol) were dissolved in DCM (10 mL) and added to round bottom flask. Then, a catalysis amount of trifluoroacetic acid (TFA) (0.3 µL, 5% mmol) were added in. The combined mixture was stirred vigorously at room temperature for overnight. After that, the reaction was monitored by TLC with additional a small amount of racemic diaminocyclohexane (CHDA). When the raw materials were consumed, the mixture was precipitated by MeOH 50 mL. The formed white precipitate was filtered and dried in vacuum to afford dry white solid molecular cage **4*MP*-HTMC**. (100 mg, 91%). ^{1}H NMR (600 MHz, $CDCl_3$), δ (ppm) = 8.36 (s, 6H), 7.79–

7.81 (d, *J* = 8.40 Hz, 12H); 7.23–7.23 (d, *J* = 8.40 Hz, 12H), 7.19 (s, 6H), 7.15–7.16 (d, *J* = 8.40 Hz, 12H), 6.81–6.82 (t, 18H), 6.72–6.73 (d, *J* = 8.40 Hz, 12H), 6.35–6.36 (d, *J* = 8.40 Hz, 12H), 3.51–5.53 (m, 6H), 1.29–1.91 (m, 24H); ^{13}C (150 MHz, $CDCl_3$), δ (ppm) = 174.29, 174.09, 173.71, 159.08, 152.99, 151.65, 151.25, 141.04, 140.69, 138.58, 138.19, 134.64, 129.64, 127.23, 126.78, 124.04, 123.58, 122.37, 121.55, 121.39, 74.36, 33.20, 24.51.

3. ^{1}H and ^{13}C NMR spectra

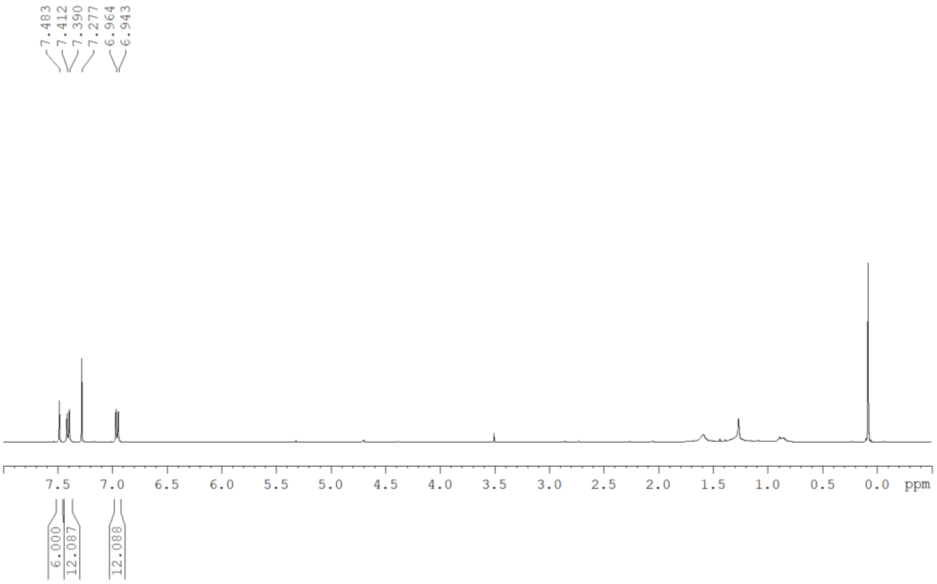

Supplementary Fig. 5. The ^{1}H NMR spectra of 1,3,5-tris(4-hydroxyphenyl)benzene based [2+3] molecular cage TMC (400 MHz, $CDCl_3$).

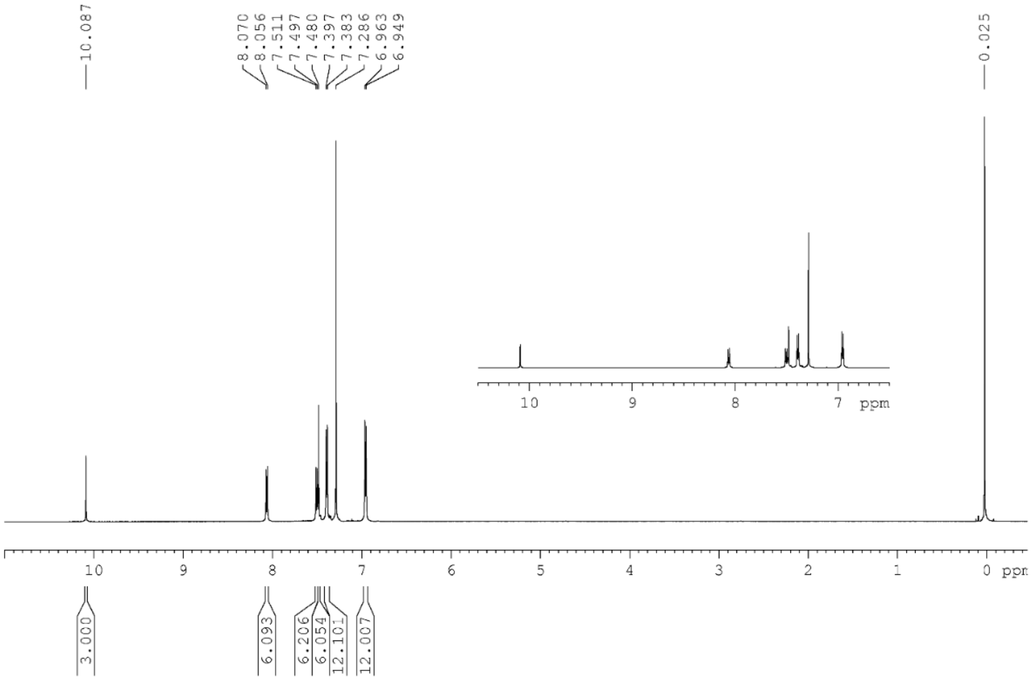

Supplementary Fig. 6. The ^{1}H NMR spectra of 1,3,5-TPB based [2+3] molecular cage CHO-TMC (600 MHz, $CDCl_3$).

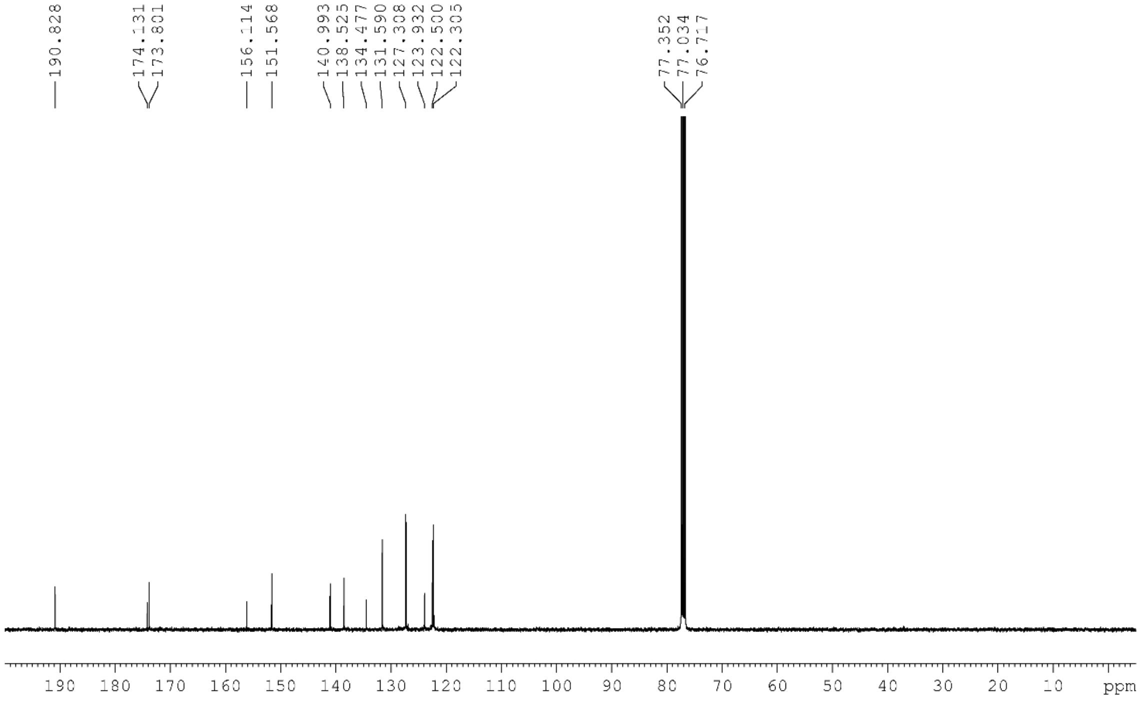

Supplementary Fig. 7. The ^{13}C NMR spectra of 1,3,5-tris(4-hydroxyphenyl)benzene based [2+3] molecular cage CHO-TMC (150 MHz, $CDCl_3$).

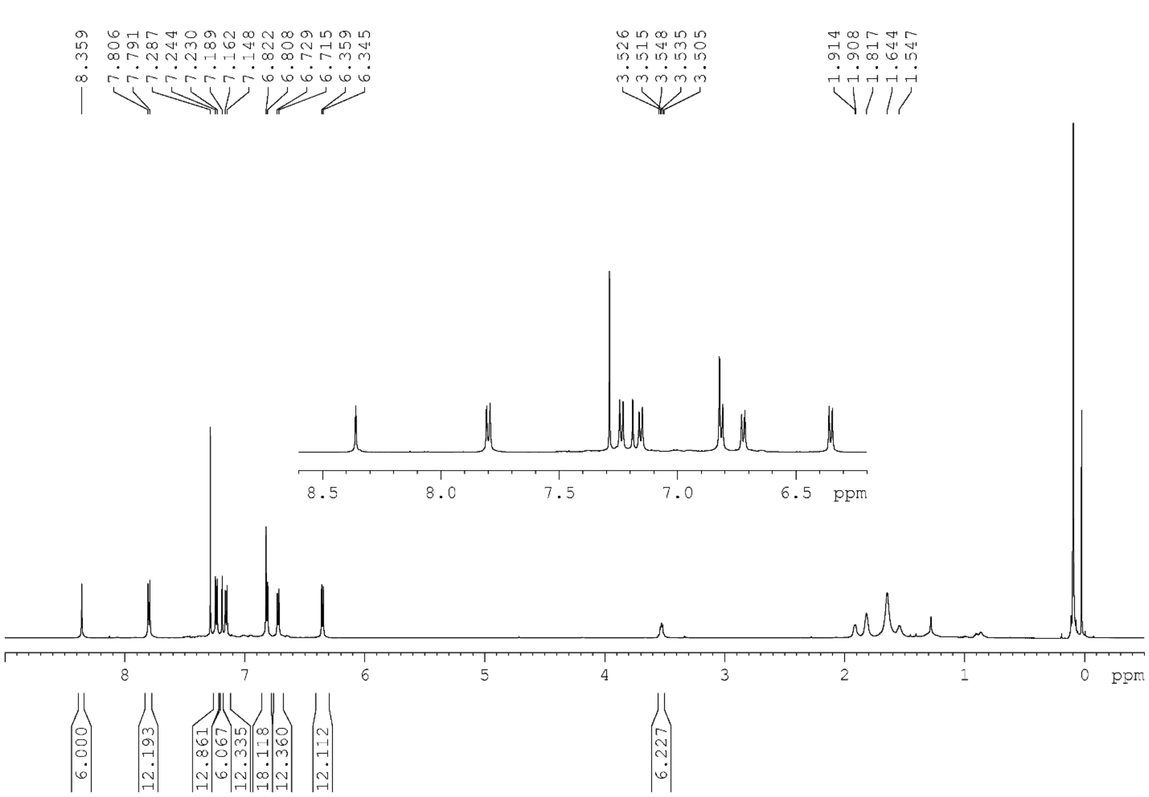

Supplementary Fig. 8. The ^{1}H NMR spectra of [2[2+3]+3] higher-level chiral molecular cage 4*P*-HTMC (600 MHz, $CDCl_3$).

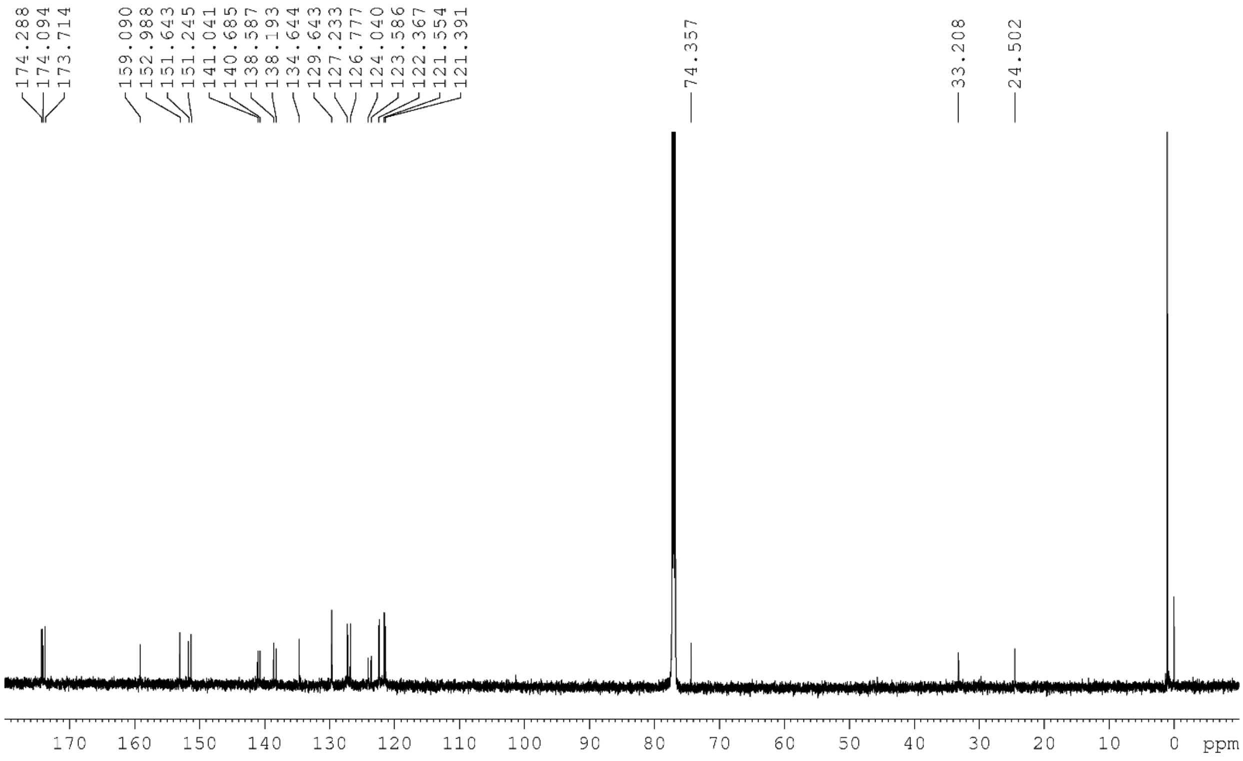

Supplementary Fig. 9. The ^{13}C NMR spectra of [2[2+3]+3] higher-level chiral molecular cage 4*P*-HTMC (150 MHz, $CDCl_3$).

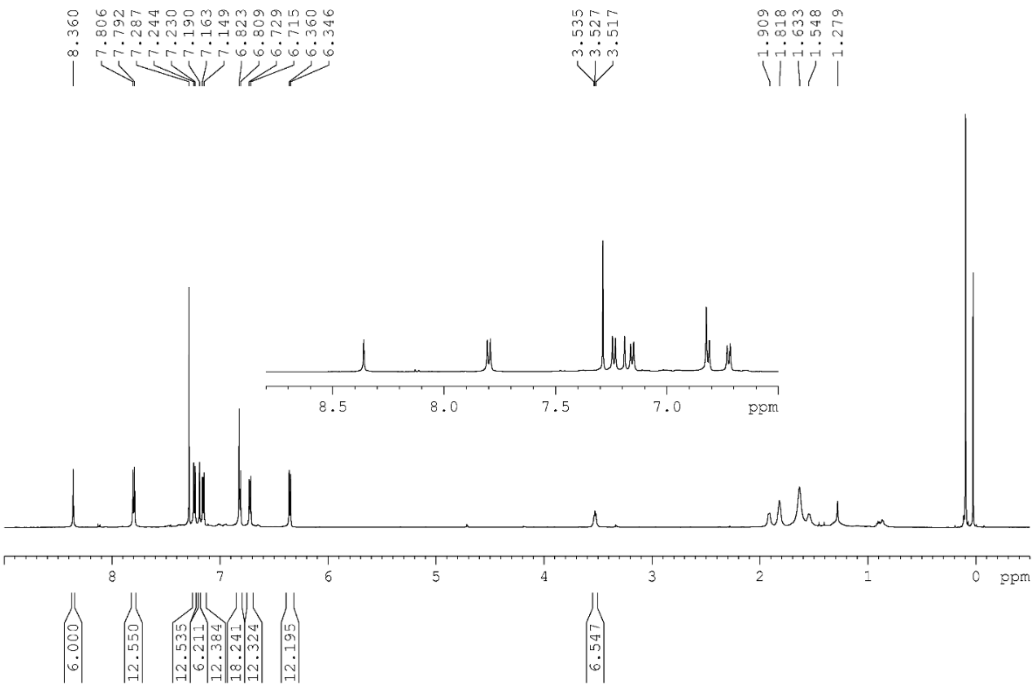

Supplementary Fig. 10. The ^{1}H NMR spectra of [2[2+3]+3] higher-level chiral molecular cage 4*M*-HTMC (600 MHz, $CDCl_3$).

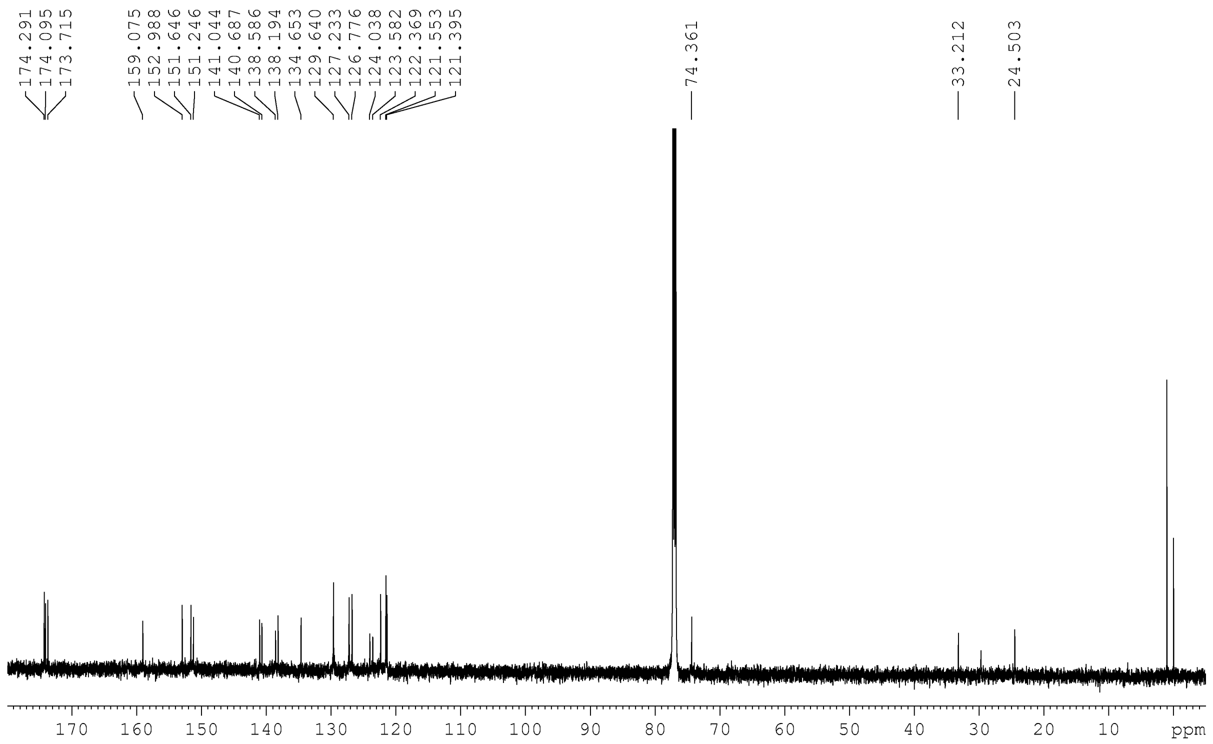

Supplementary Fig. 11. The ^{13}C NMR spectra of [2[2+3]+3] higher-level chiral molecular cage 4*M*-HTMC (150 MHz, $CDCl_3$).

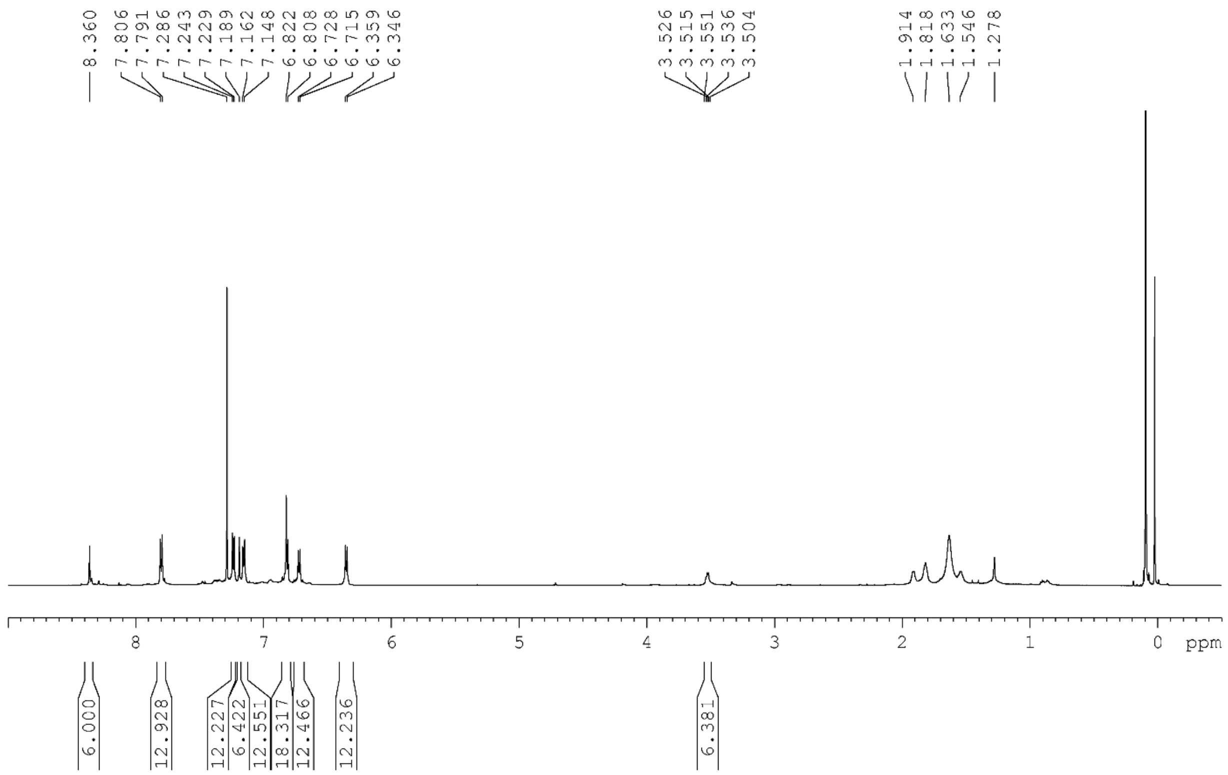

Supplementary Fig. 12. The ^{1}H NMR spectra of [2[2+3]+3] higher-level racemic molecular cage 4*MP*-HTMC (600 MHz, $CDCl_3$).

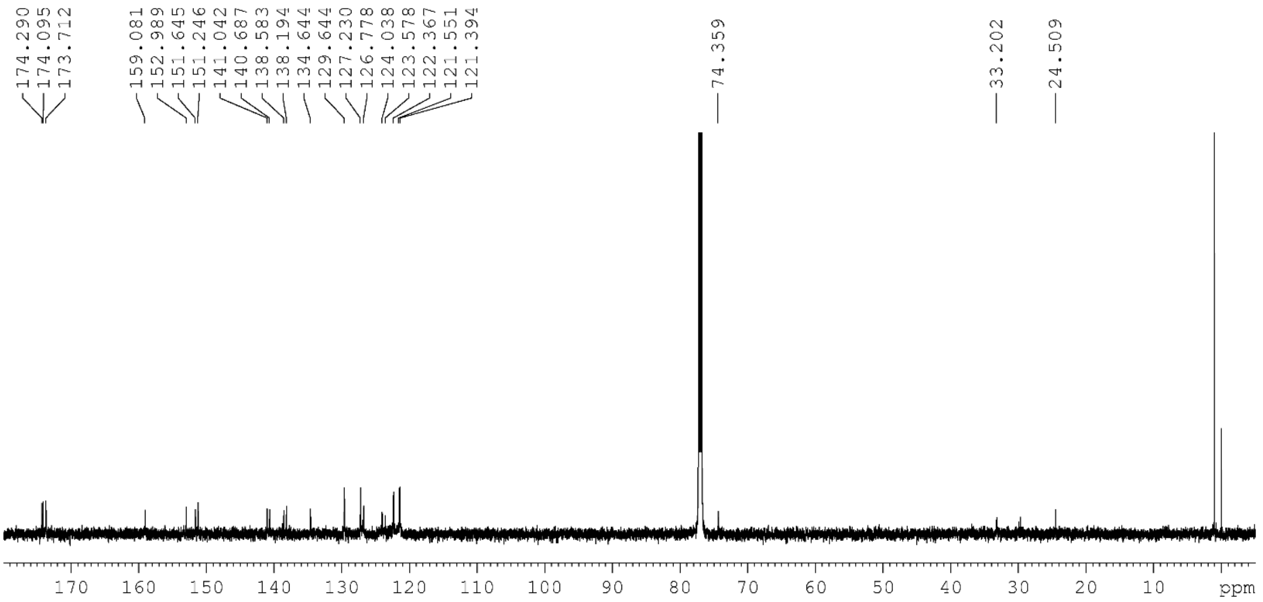

Supplementary Fig. 13. The ^{13}C NMR spectra of [2[2+3]+3] higher-level racemic molecular cage 4*MP*-HTMC (150 MHz, $CDCl_3$).

4. 2D ^{1}H,^{1}H-COSY, ^{1}H,^{1}H-NOESY, ^{1}H,^{13}C-HSQC and ^{1}H,^{13}C-HMBC NMR spectra

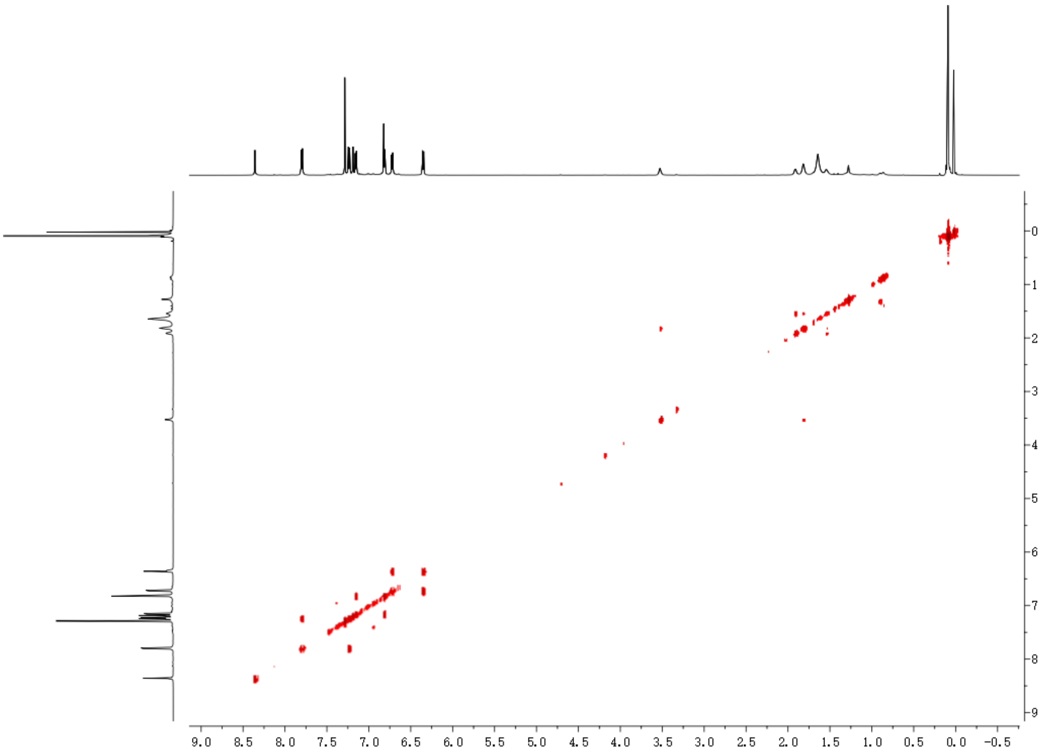

Supplementary Fig. 14. The ^{1}H,^{1}H-COSY spectra of [2[2+3]+3] higher-level chiral molecular cage 4*P*-HTMC (600 MHz, $CDCl_3$).

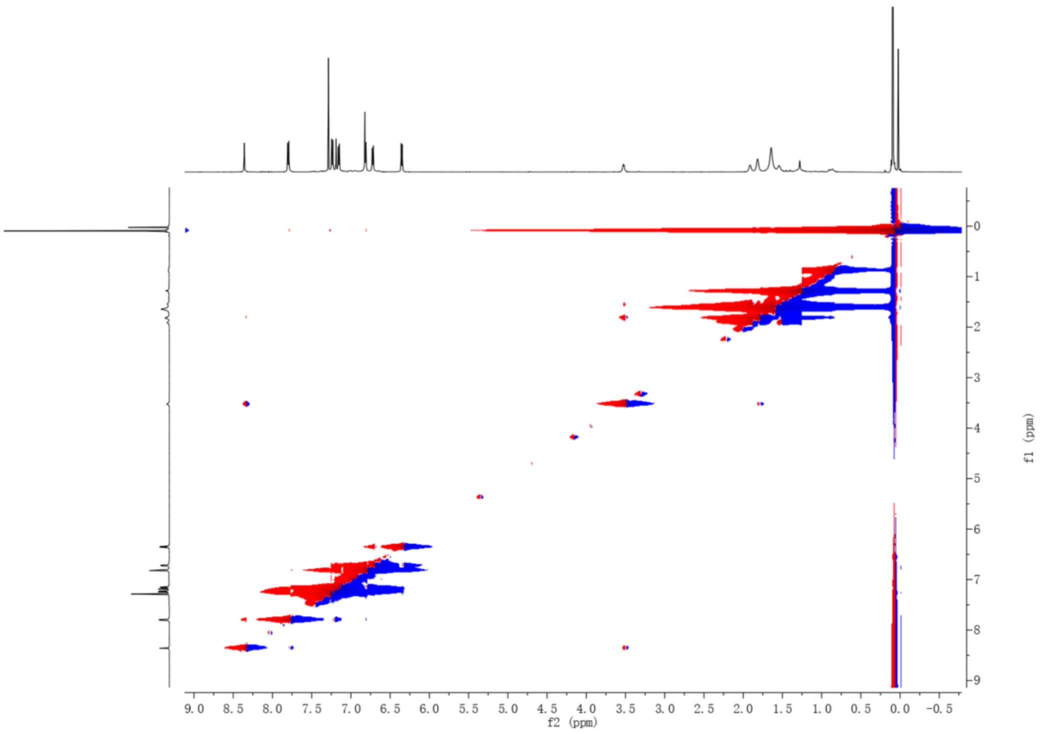

Supplementary Fig. 15. The ^{1}H,^{1}H- NOESY spectra of [2[2+3]+3] higher-level chiral molecular cage 4*P*-HTMC (600 MHz, $CDCl_3$).

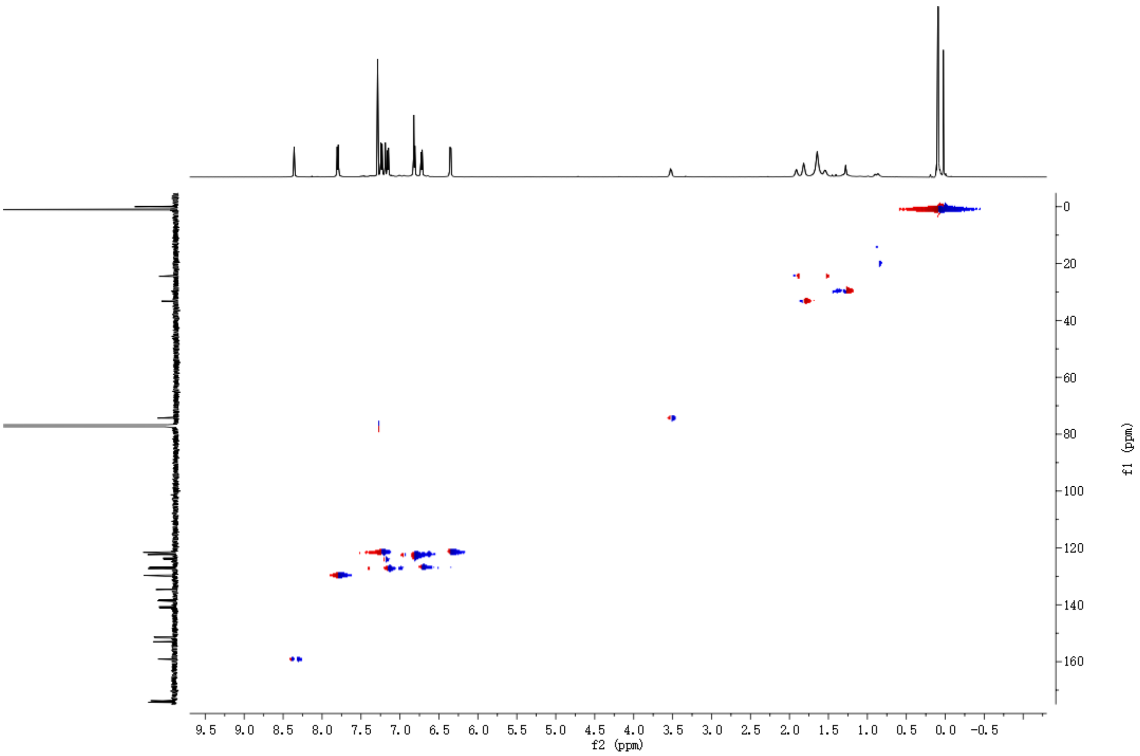

Supplementary Fig. 16. The ^{1}H,^{13}C- HSQC spectra of [2[2+3]+3] higher-level chiral molecular cage 4*P*-HTMC (600 MHz and 150 MHz, $CDCl_3$).

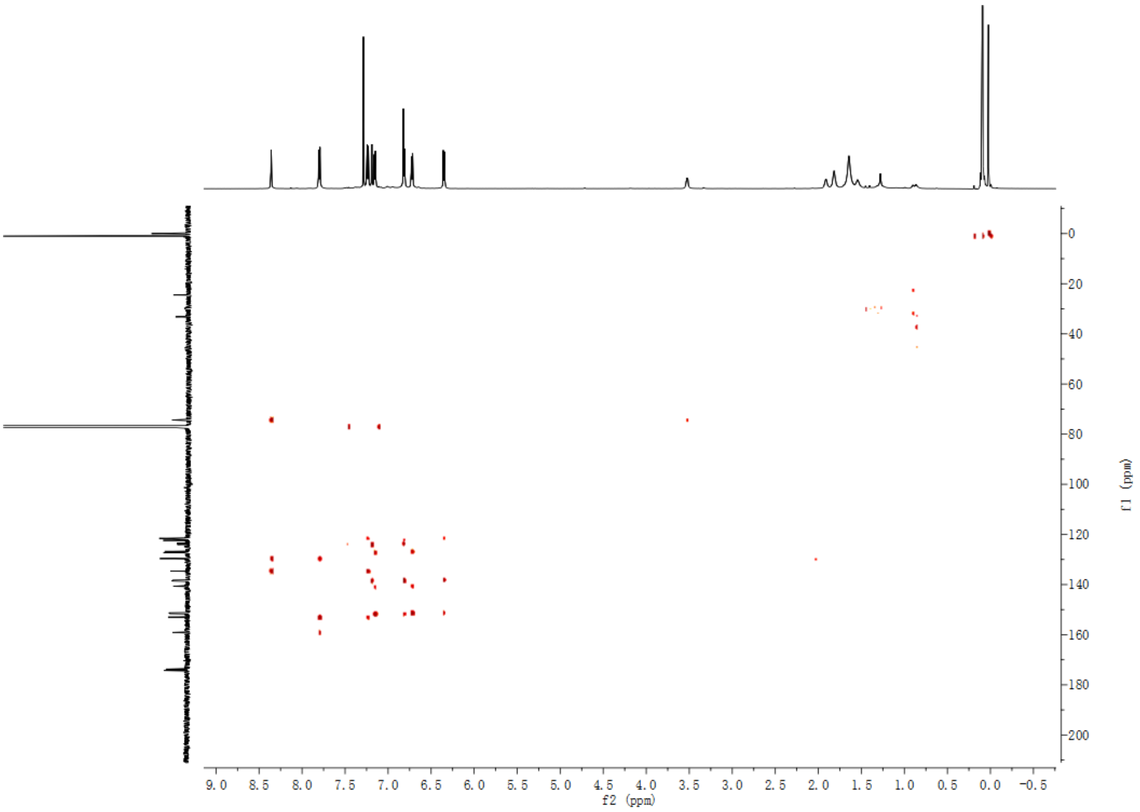

Supplementary Fig. 17. The ^{1}H,^{13}C-HMBC spectra of [2[2+3]+3] higher-level chiral molecular cage 4*P*-HTMC (600 MHz and 150 MHz, $CDCl_3$).

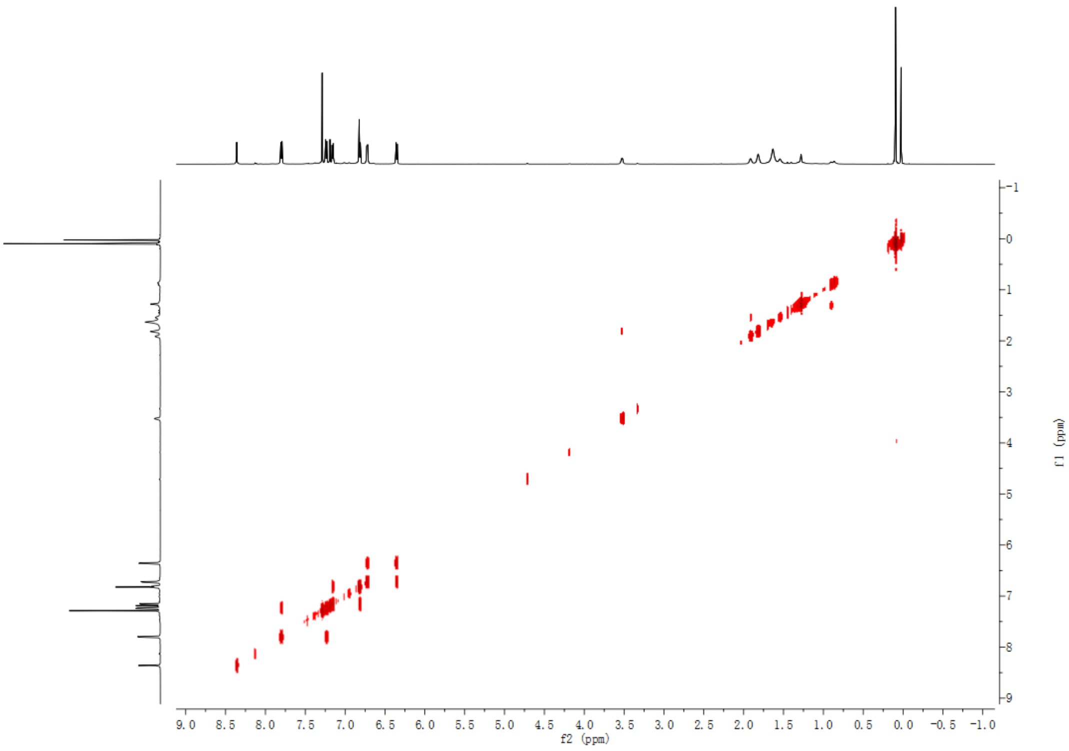

Supplementary Fig. 18. The ^{1}H,^{1}H-COSY spectra of [2[2+3]+3] higher-level chiral molecular cage 4*M*-HTMC (600 MHz, $CDCl_3$).

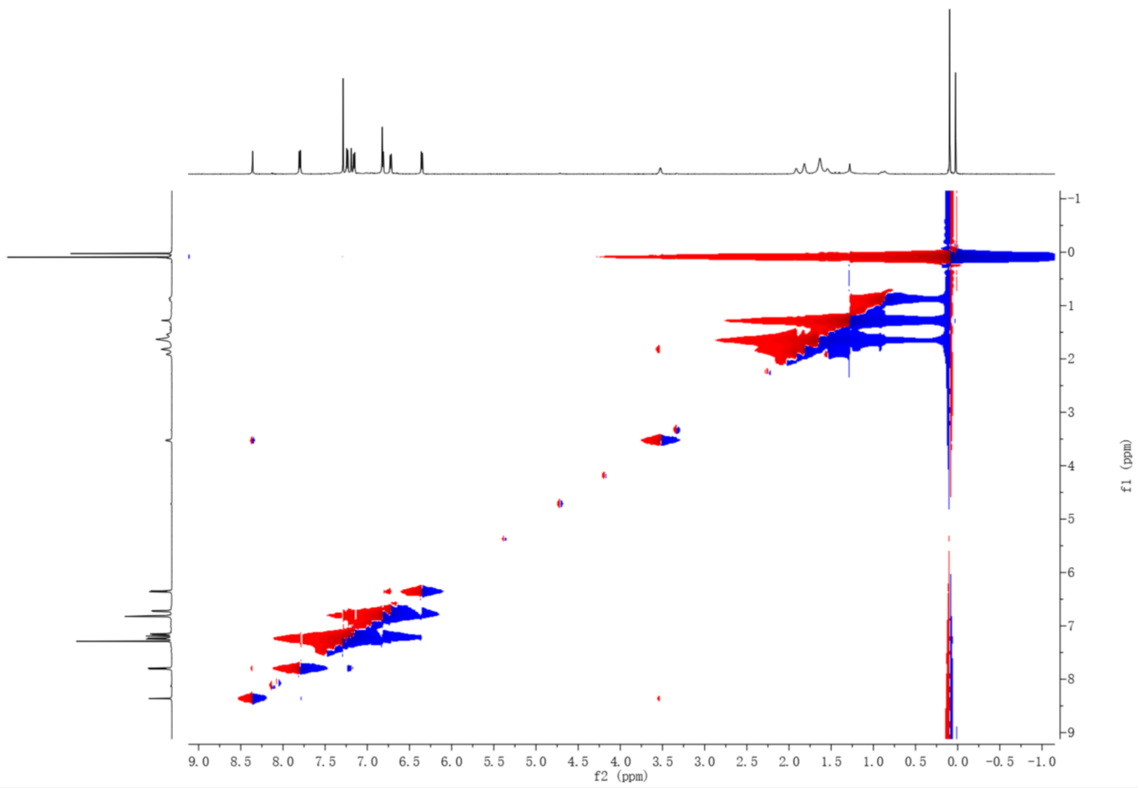

Supplementary Fig. 19. The $^{1}H,^{1}H$- NOESY spectra of [2[2+3]+3] higher-level chiral molecular cage 4*M*-HTMC (600 MHz, $CDCl_3$).

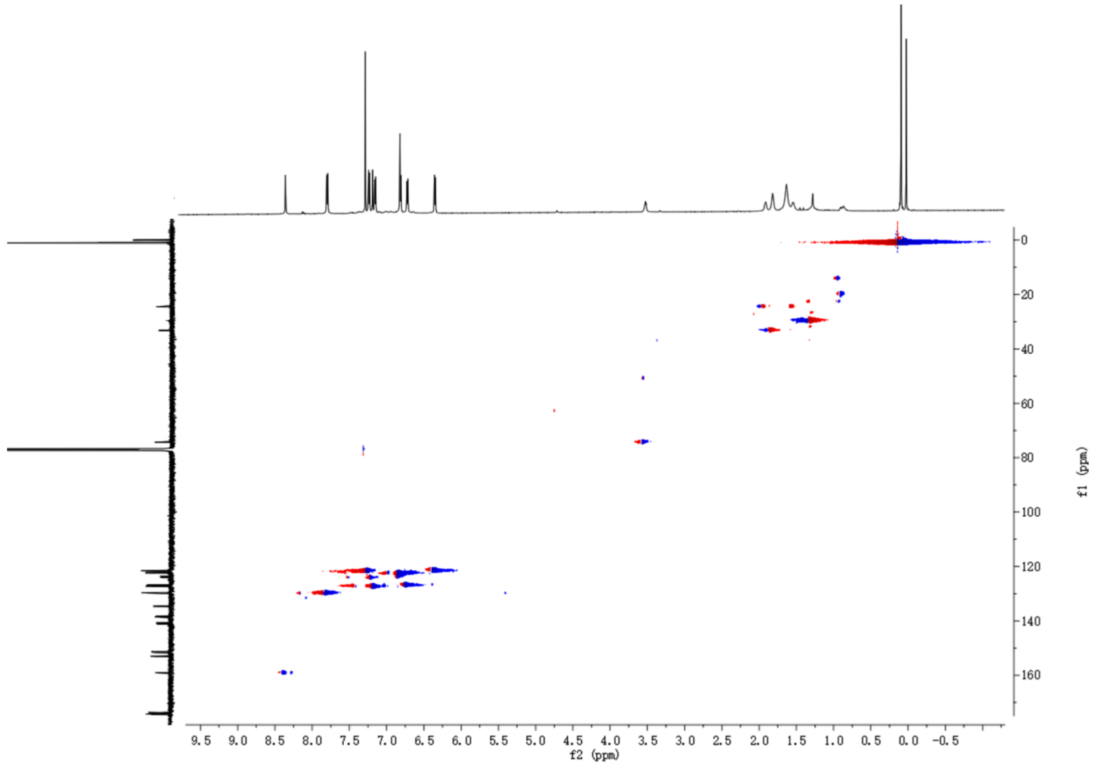

Supplementary Fig. 20. The $^{1}H,^{13}C$- HSQC spectra of [2[2+3]+3] higher-level chiral molecular cage 4*M*-HTMC (600 MHz and 150 MHz, $CDCl_3$).

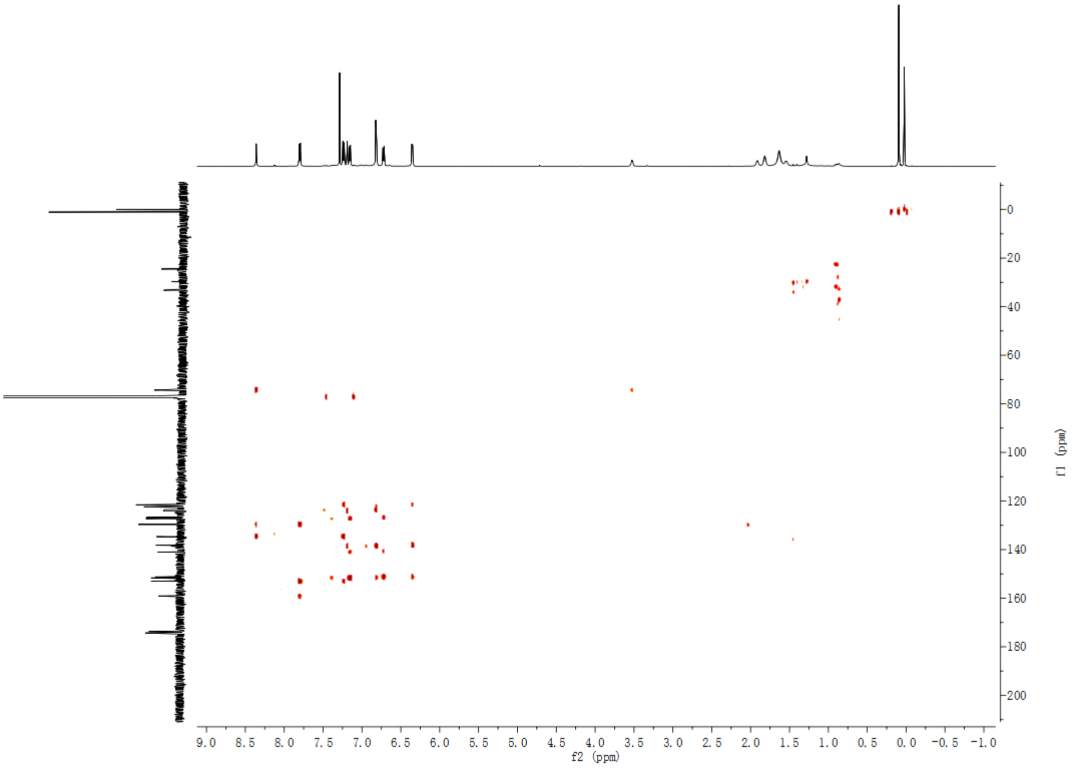

Supplementary Fig. 21. The ^{1}H,^{13}C-HMBC spectra of [2[2+3]+3] higher-level chiral molecular cage 4*M*-HTMC (600 MHz and 150 MHz, $CDCl_3$).

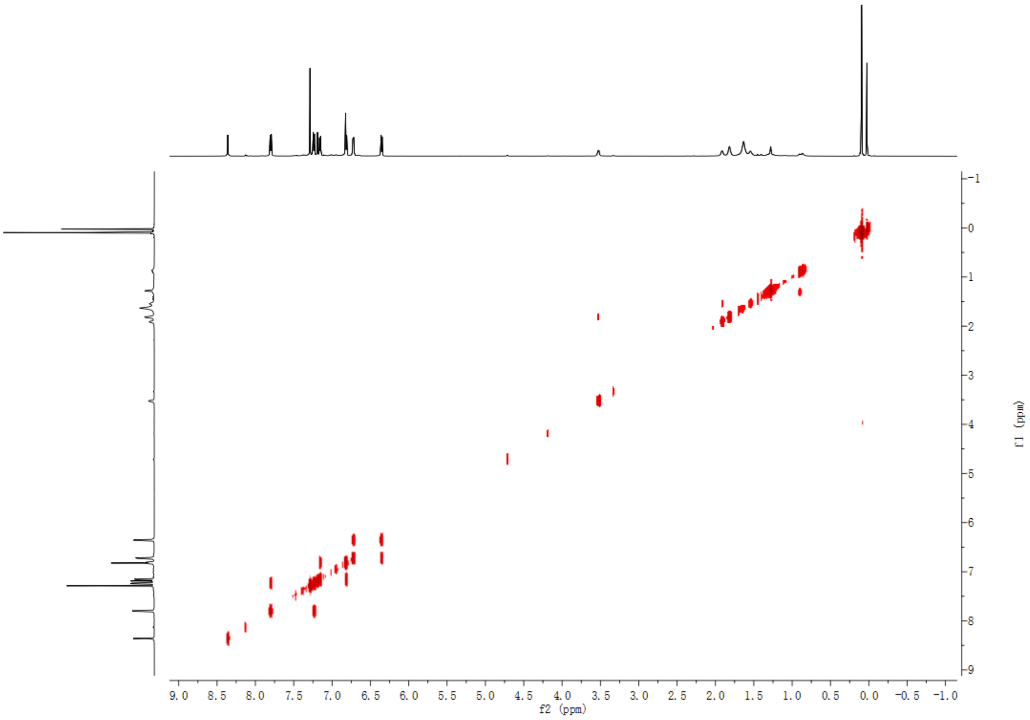

Supplementary Fig. 22. The ^{1}H,^{1}H-COSY spectra of [2[2+3]+3] higher-level racemic molecular cage 4*MP*-HTMC (600 MHz, $CDCl_3$).

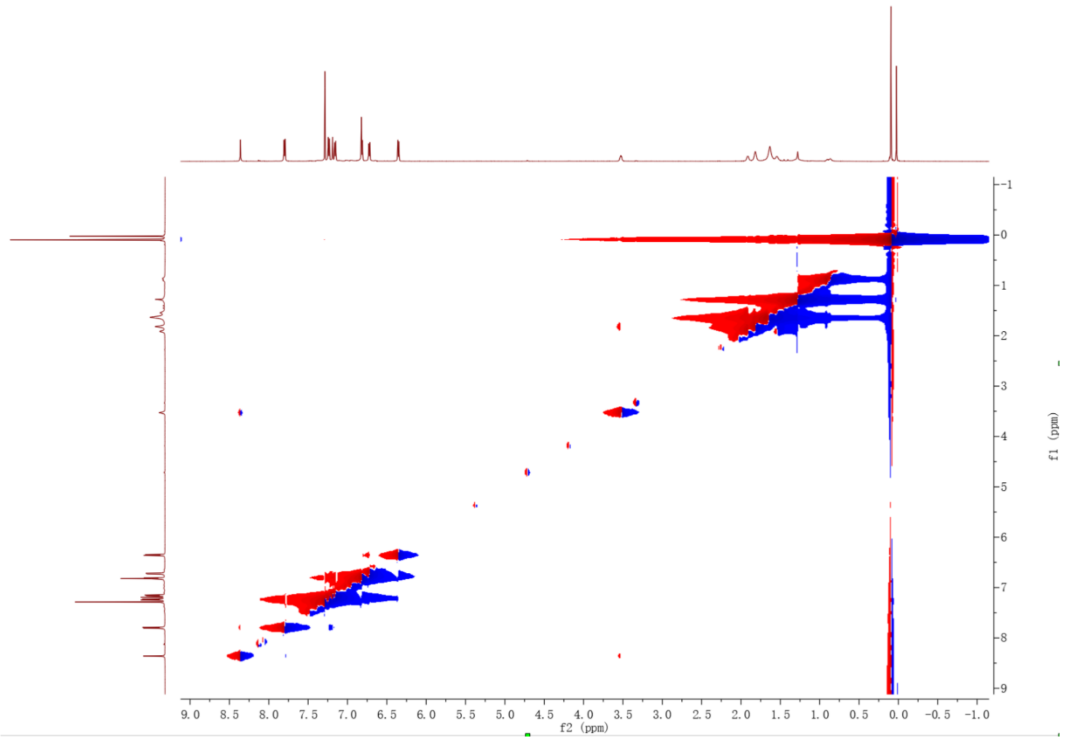

Supplementary Fig. 23. The ^{1}H,^{1}H- NOESY spectra of [2[2+3]+3] higher-level racemic molecular cage 4*MP*-HTMC (600 MHz, $CDCl_3$).

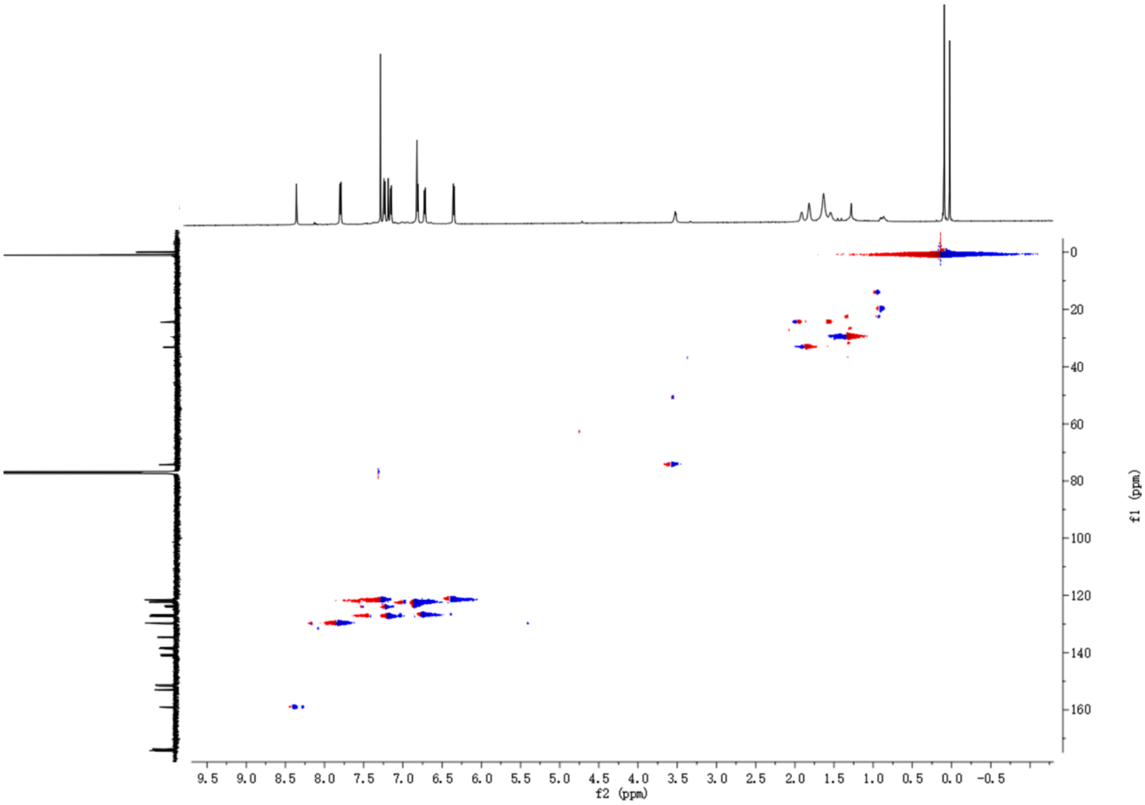

Supplementary Fig. 24. The ^{1}H,^{13}C- HSQC spectra of [2[2+3]+3] higher-level racemic molecular cage 4*MP*-HTMC (600 MHz and 150 MHz, $CDCl_3$).

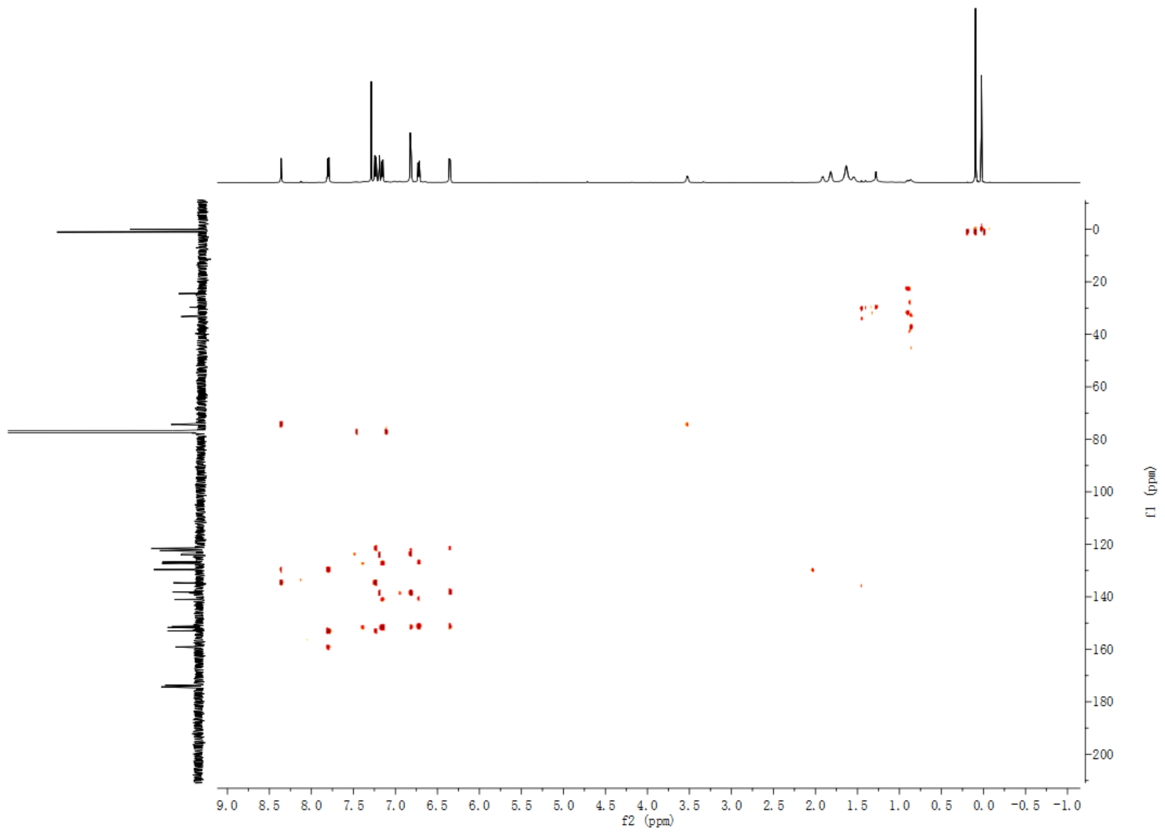

Supplementary Fig. 25. The ^{1}H,^{13}C-HMBC spectra of [2[2+3]+3] higher-level racemic molecular cage 4*MP*-HTMC (600 MHz and 150 MHz, $CDCl_3$).

5. ^{1}H NMR titration.

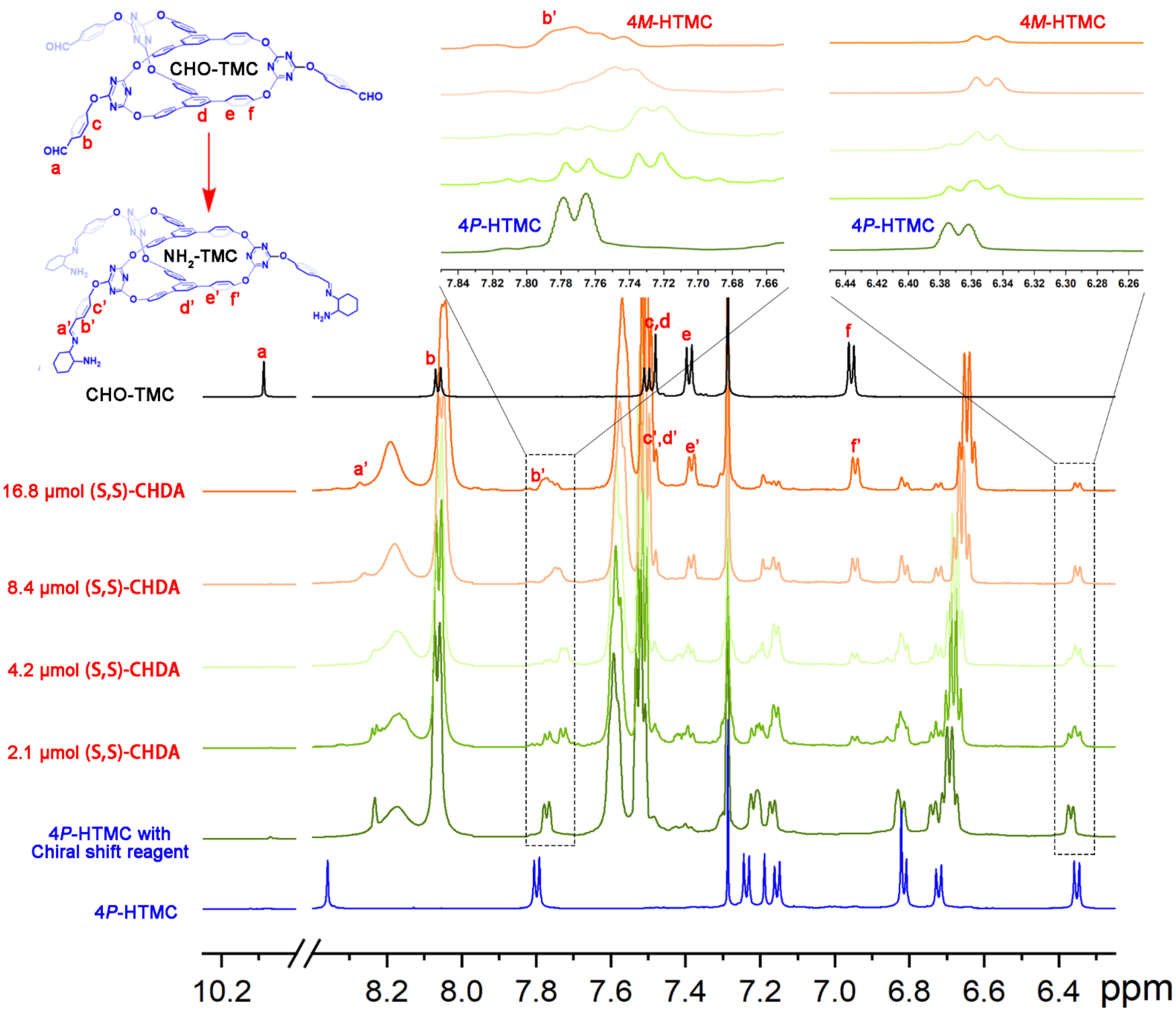

Supplementary Fig. 26. The ^{1}H NMR (600 MHz, $CDCl_3$) of molecular cages 4*P*-HTMC with an excess of the chiral shift reagent (*S*)-(+)-2,2,2-trifluoro-1-(9-anthryl)ethanol in $CDCl_3$ upon adding different quantity of (*S*,*S*)-diaminocyclohexane CHDA.

6. MALDI-TOF mass spectrometry

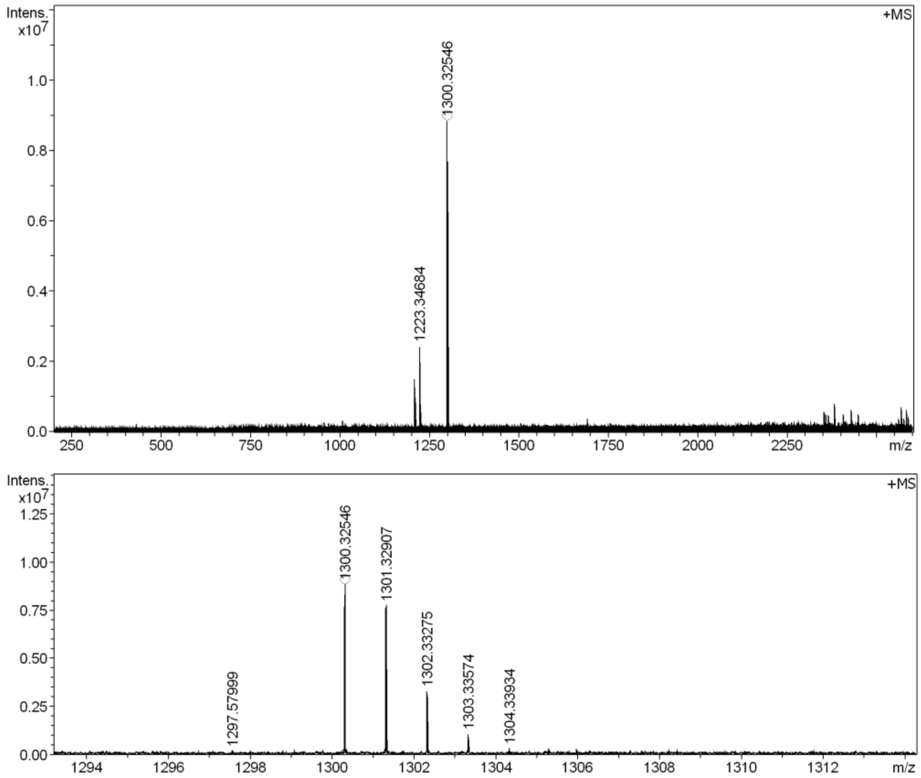

Supplementary Fig. 27. The MALDI-TOF mass spectrometry of CHO-TMC.

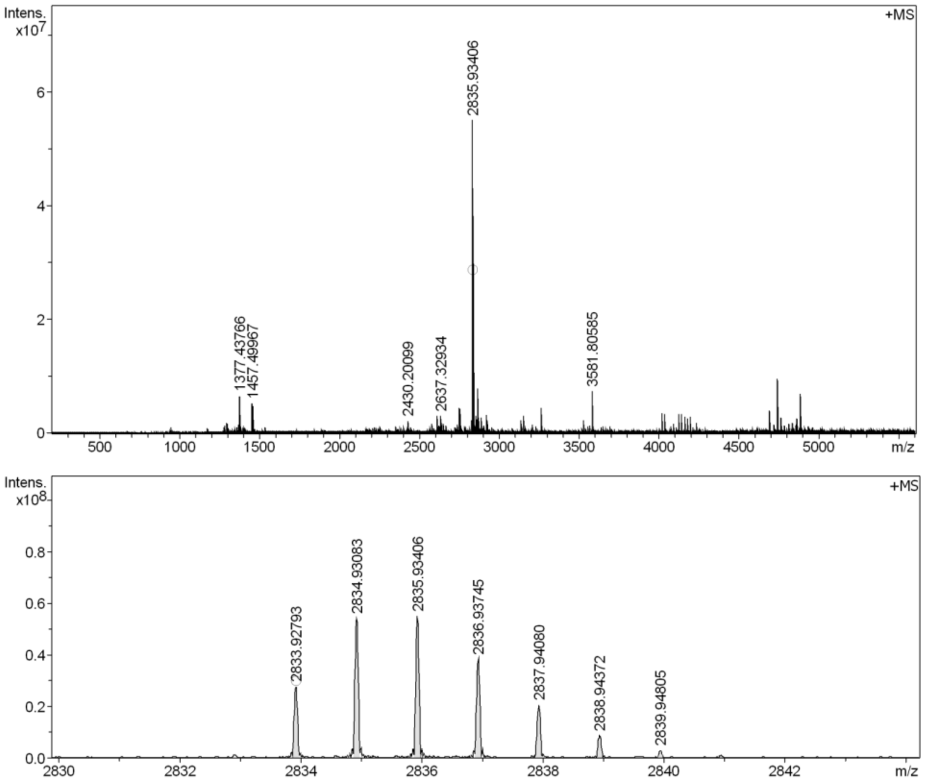

Supplementary Fig. 28. The MALDI-TOF mass spectrometry of 4*P*-HTMC.

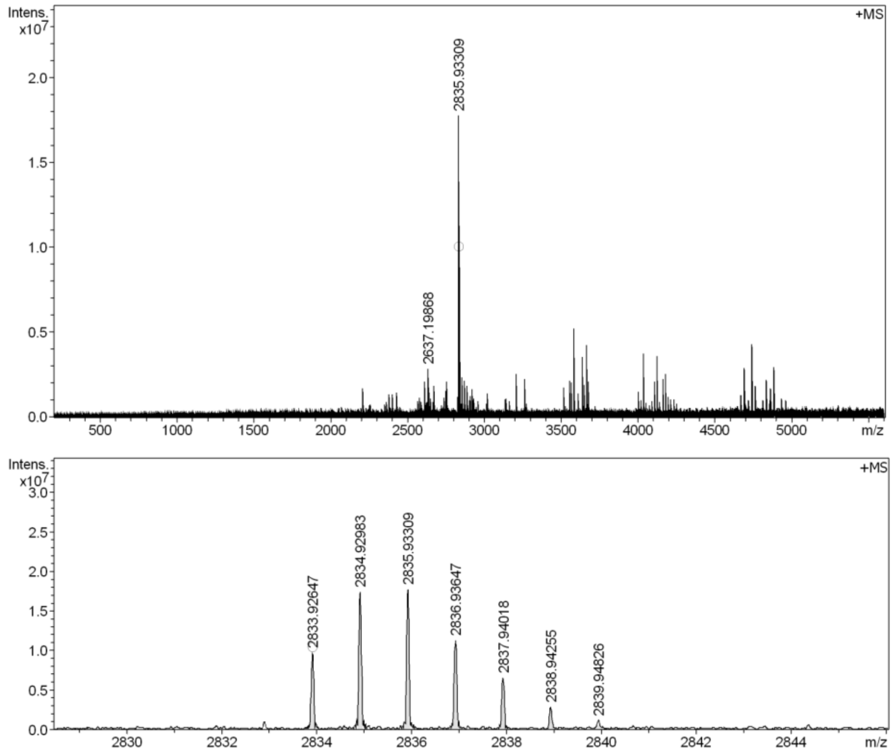

Supplementary Fig. 29. The MALDI-TOF mass spectrometry of 4*M*-HTMC.

7. X-ray single crystal diffraction structures.

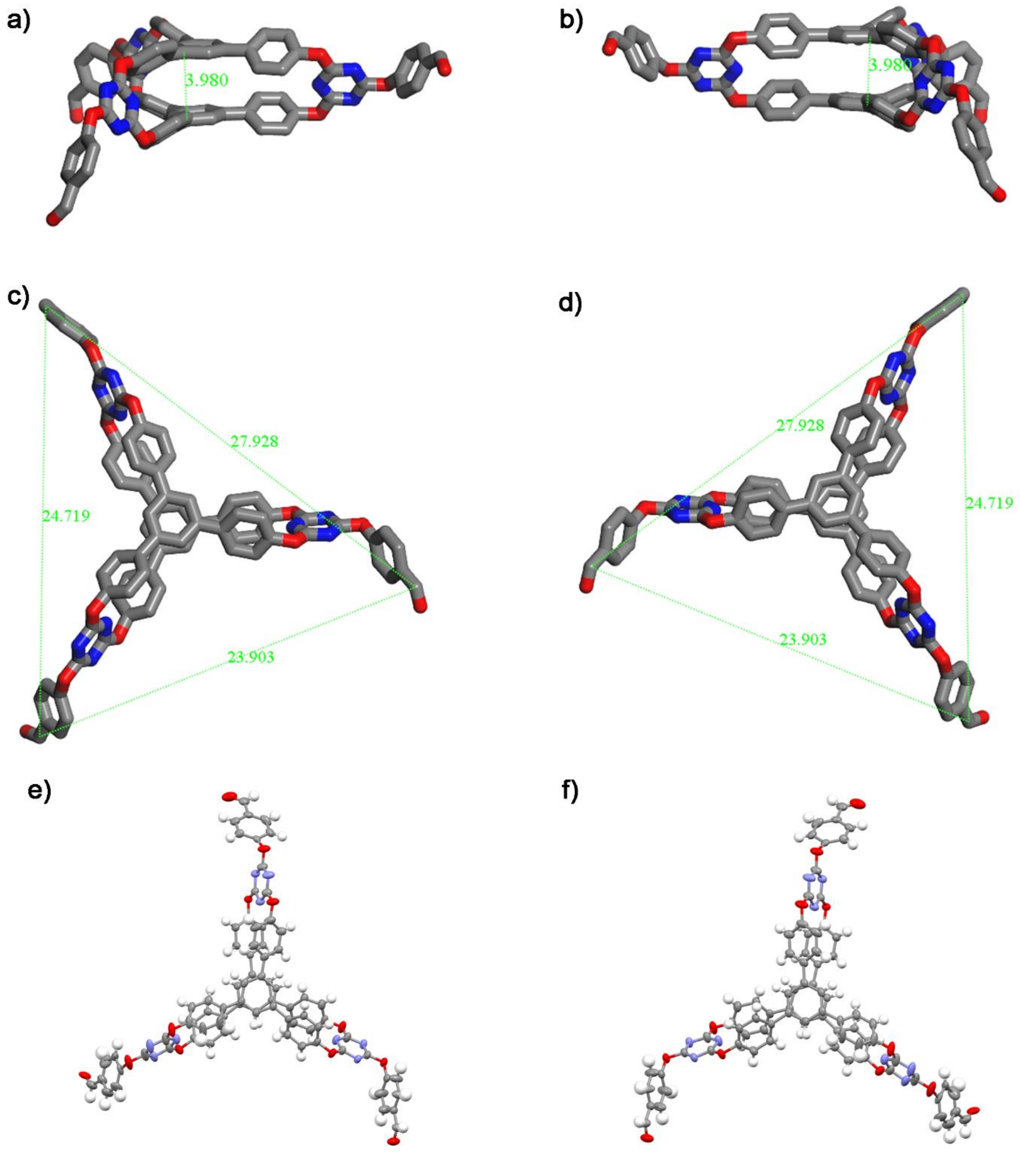

Supplementary Fig. 30. The chemical structure and X-ray single crystal structures from side view (a and b) and top view (c and d) molecular cages 2*P*-CHO-TMC and 2*M*-CHO-TMC, respectively. The cavity size of them was around 3.98 Å determined by the distance of central phenyl rings. (Hydrogen atom was omitted for clarity). The Oak Ridge thermal ellipsoid plot ORTEP-style illustration of molecular cages (e) 2*P*-CHO-TMC and (f) 2*M*-CHO-TMC (CCDC: 2180996).

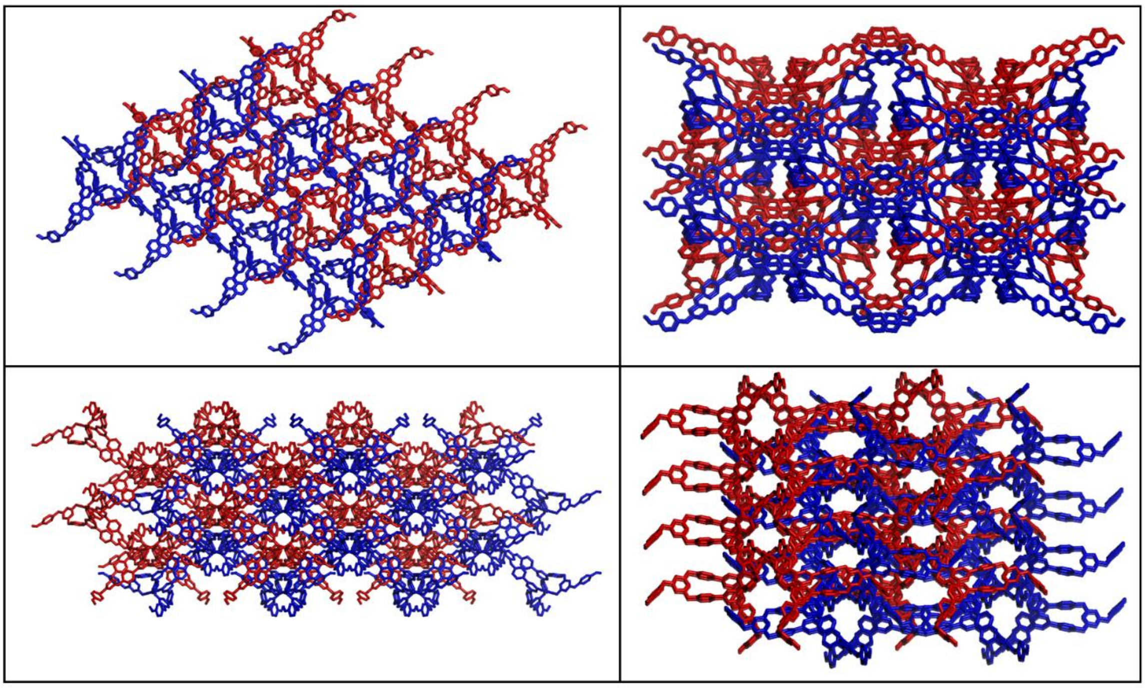

Supplementary Fig. 31. The assemble structures from different view perspectives of molecular cages 2*P*-CHO-TMC (blue) and 2*M*-CHO-TMC (red) analyzed by the X-ray single crystal structures of CHO-TMC. The enantiomers molecular cages 2*P*-CHO-TMC (blue) and 2*M*-CHO-TMC (red) coexist in equal proportions with intertwined network structures. (Hydrogen atom was omitted for clarity).

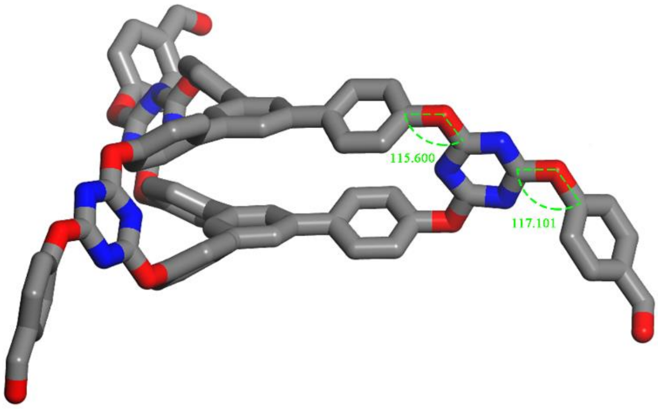

Supplementary Fig. 32. The X-ray single crystal structures of molecular cage CHO-TMC shows similar C-O-C bonds.

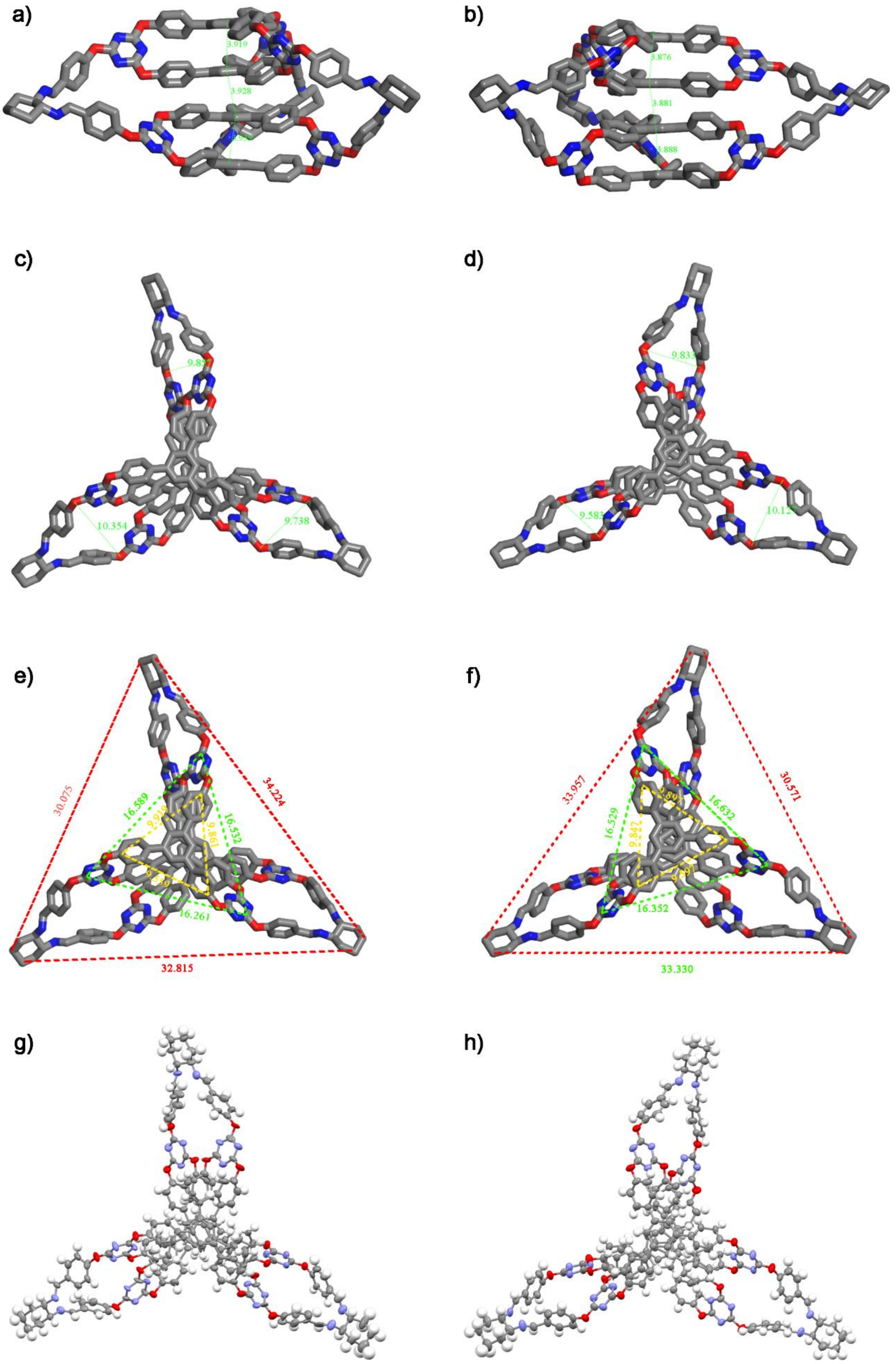

Supplementary Fig. 33. The X-ray single crystal structures of molecular cages 4*P*-HTMC (left) and 4*M*-HTMC (right) from side view (a and b) show cavities size was around 3.9 Å determined by the distance of central phenyl rings. The top view

shows the hetero-pores around 10 Å (c and d) and the molecular size of 4*P*-HTMC and 4*M*-HTMC, the building blocks CHO-TMC () and building blocks' building blocks TPB are about 30-34 Å, 16 Å and 10 Å, determined by the vertices distance of the tri-bladed propeller structures (e and f). (Hydrogen atom was omitted for clarity). The Oak Ridge thermal ellipsoid plot ORTEP-style illustration of molecular cages (g) 4*P*-HTMC (CCDC: 2181020) and (h) and 4*M*-HTMC (CCDC: 2181024).

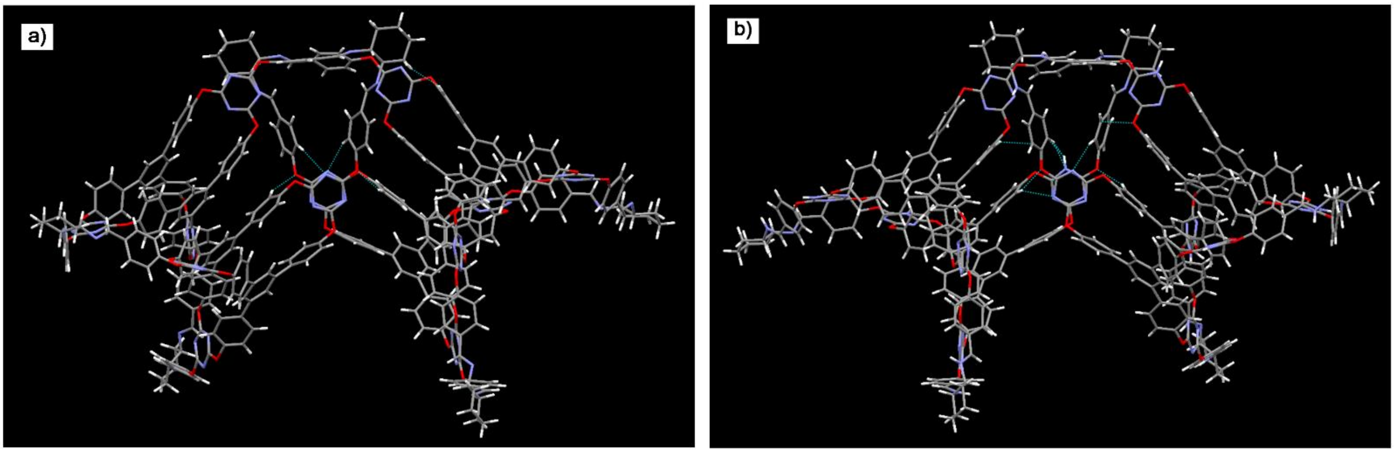

Supplementary Fig. 34. The X-ray single crystal structures of molecular cages 4*P*-HTMC (a) and 4*M*-HTMC (b) that assemble into dimers by intermolecular C−H···N interactions occur between the aromatic protons and the nitrogen atom of the triazine ring, and the π-π stacking interactions take place between the triazine ring moieties of adjacent molecular cages.

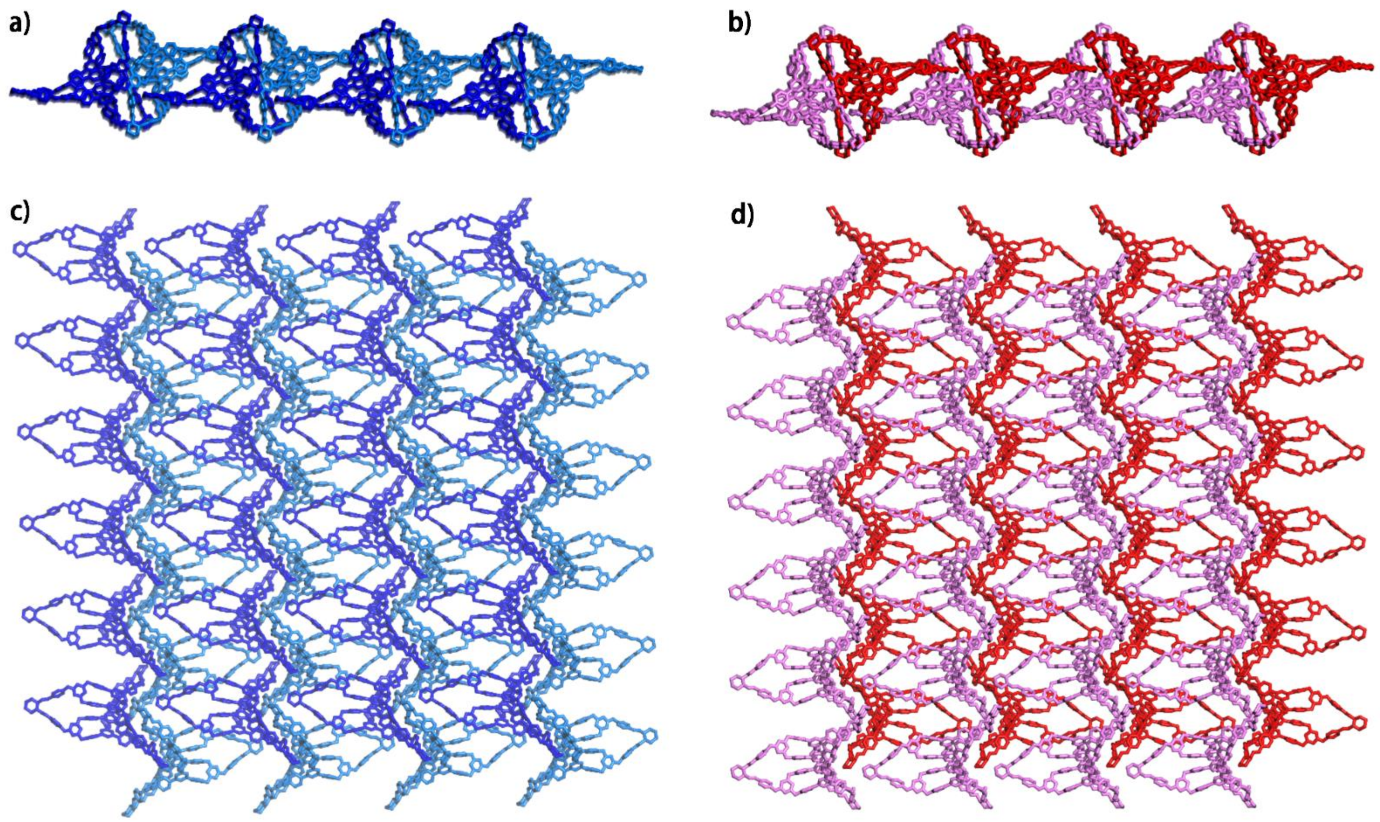

Supplementary Fig. 35. The assemble structures of molecular cages 4*P*-HTMC (blue) and 4*M*-HTMC (red) analyzed by the X-ray single crystal structures of CHO-TMC. Through intermolecular interactions, the formed neighboring helices of molecular cages (a) 4*P*-HTMC (blue) and (b) 4*M*-HTMC (red) align and stack together, resulting in the formation of 2D layered structures (c) 4*P*-HTMC (blue) and (d) 4*M*-HTMC. (Hydrogen atom was omitted for clarity).

8. Computational calculation.

The total energy calculation for molecular cages 2*P*-CHO-TMC and 2*M*-CHO-TMC and all four possible isomers HTMC with different ratio of (*R*,*R*)-(CHDA)/(*S*,*S*)-(CHDA) (R_3, S_3, R_2S and RS_2) were performed with Material Studio 8.0 using *DMol3* model. The task was set to *Geometry Optimization* with Quality of *Medium* and Functional of *GGA* and *BLYP*.

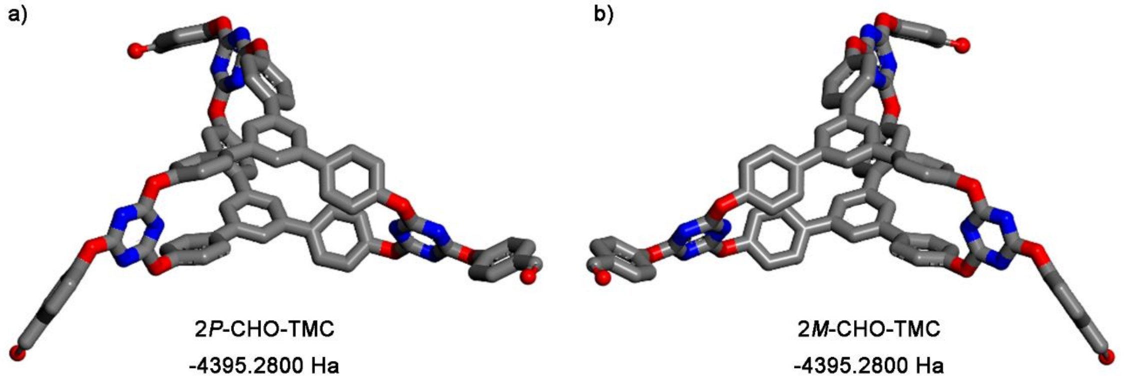

Supplementary Fig. 36. The optimized chemical structure and their total energy of four possible molecular cages (a) 2*P*-CHO-TMC and (b) 2*M*-CHO-TMC after *Geometry Optmization*. (Hydrogen atom was omitted for clarity).

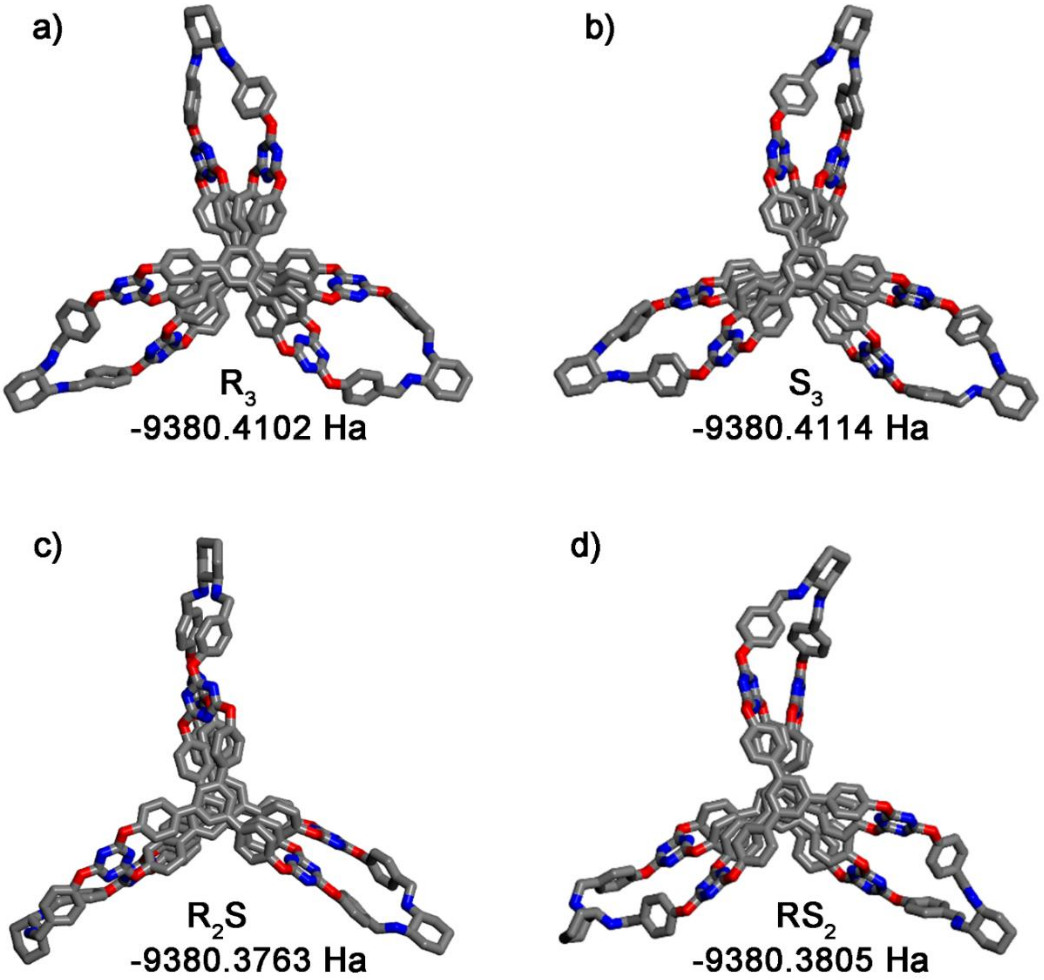

Supplementary Fig. 37. The optimized chemical structure and their total energy of four possible isomers HTMC with different ratio of (*R*,*R*)-(CHDA)/(*S*,*S*)-(CHDA) (a) R_3, (b) S_3, (c) R_2S and (d) RS_2 after *Geometry Optmization*. (Hydrogen atom was omitted for clarity).

9. UV absorption spectra and Circular dichroism (CD) spectra.

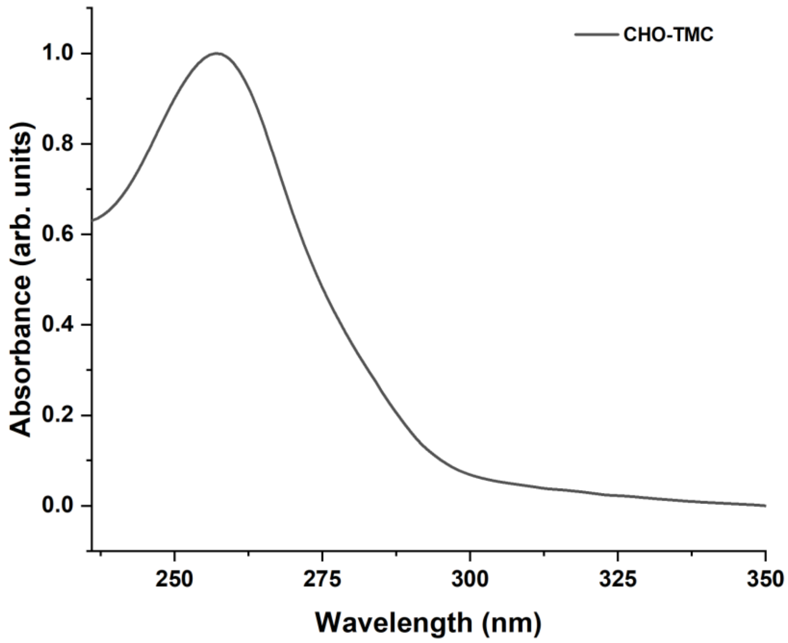

Supplementary Fig. 38. The UV absorption spectra of molecular cage CHO-TMC in DCM (*c* = 0.5 mM).

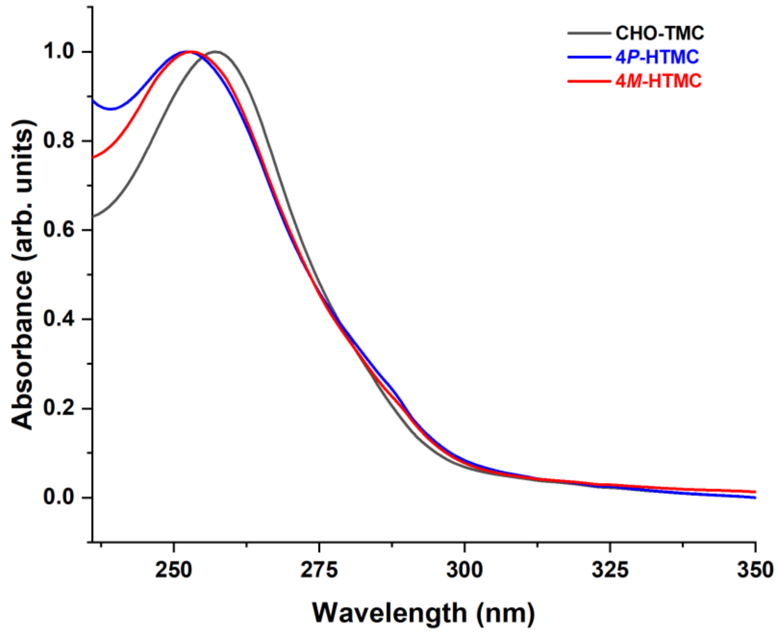

Supplementary Fig. 39. The UV absorption spectra of molecular cage CHO-TMC, 4*P*-HTMC and 4*M*-HTMC in DCM (*c* = 0.5 mM).

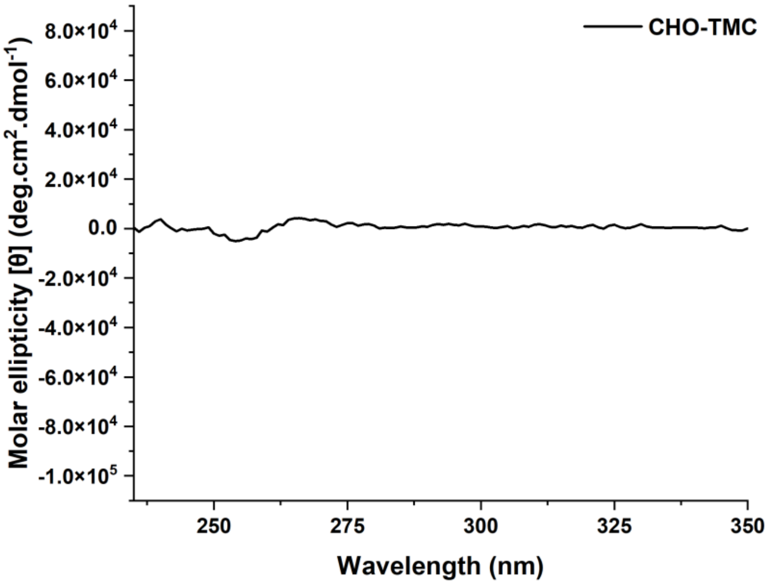

Supplementary Fig. 40. The CD spectra of molecular cage CHO-TMC in DCM (c = 0.5 mM).

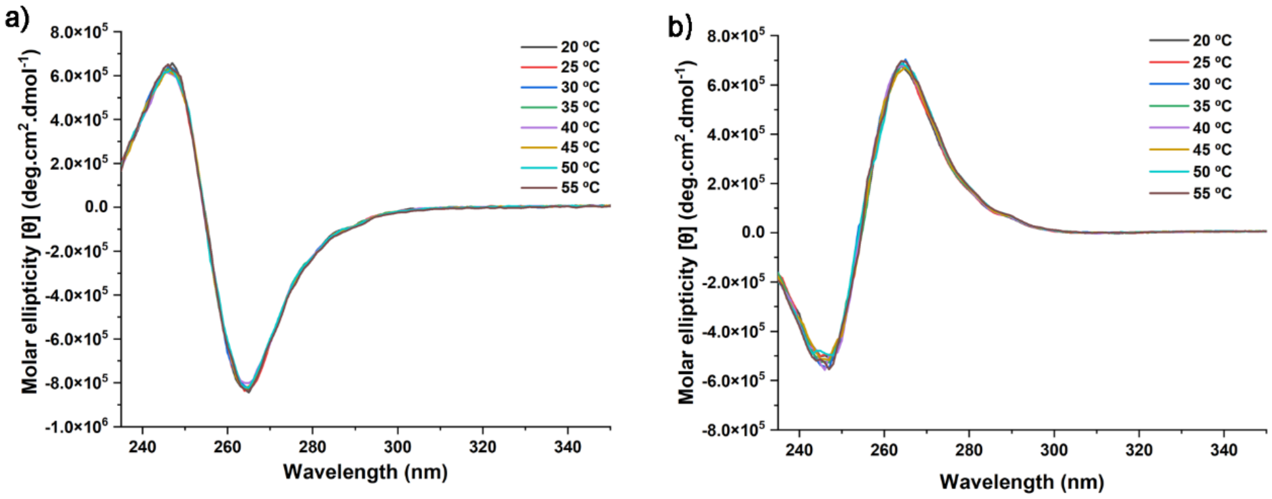

Supplementary Fig. 41. The temperature variable CD spectra of molecular cage 4*P*-HTMC (a) and 4*M*-HTMC (b) at different 20, 25, 30, 35, 40, 45, 50, 55 °C.

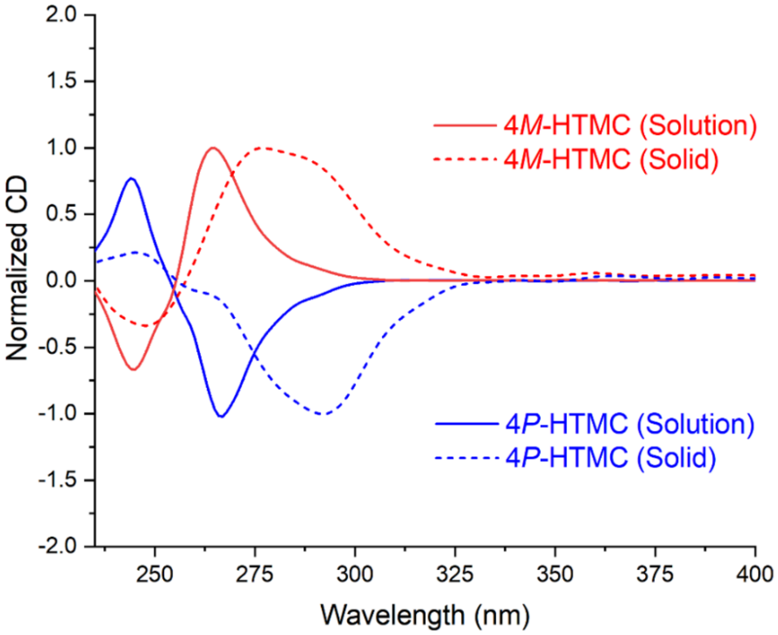

Supplementary Fig. 42. The CD spectra of molecular cage 4*P*-HTMC (blue) and 4*M*-HTMC (red) in dichloromethane DCM (c = 0.5 mM) and in solid state.

10.SEM and TEM.

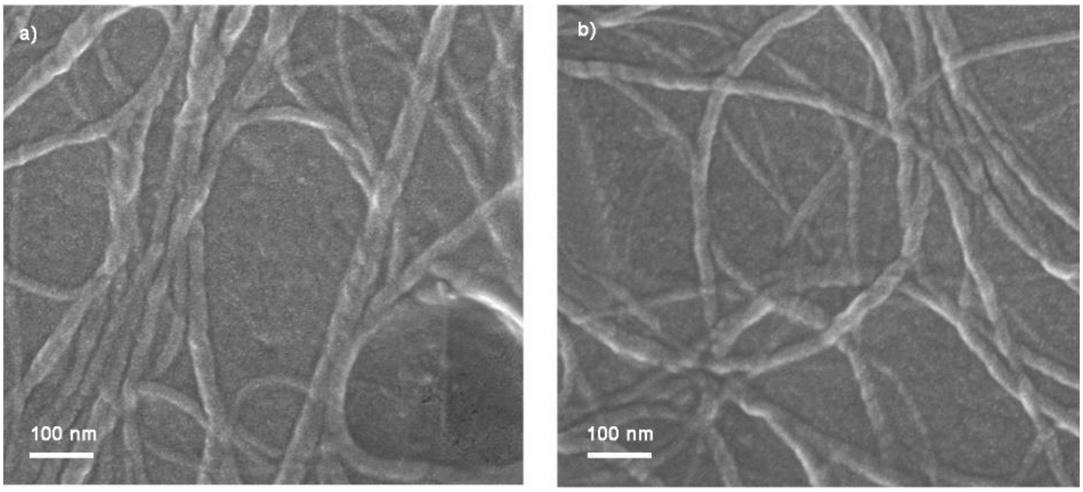

Supplementary Fig. 43. The SEM images of d) molecular cage 4*P*-HTMC and f) 4*M*-HTMC assembled L-helical or D-helical nanofibers.

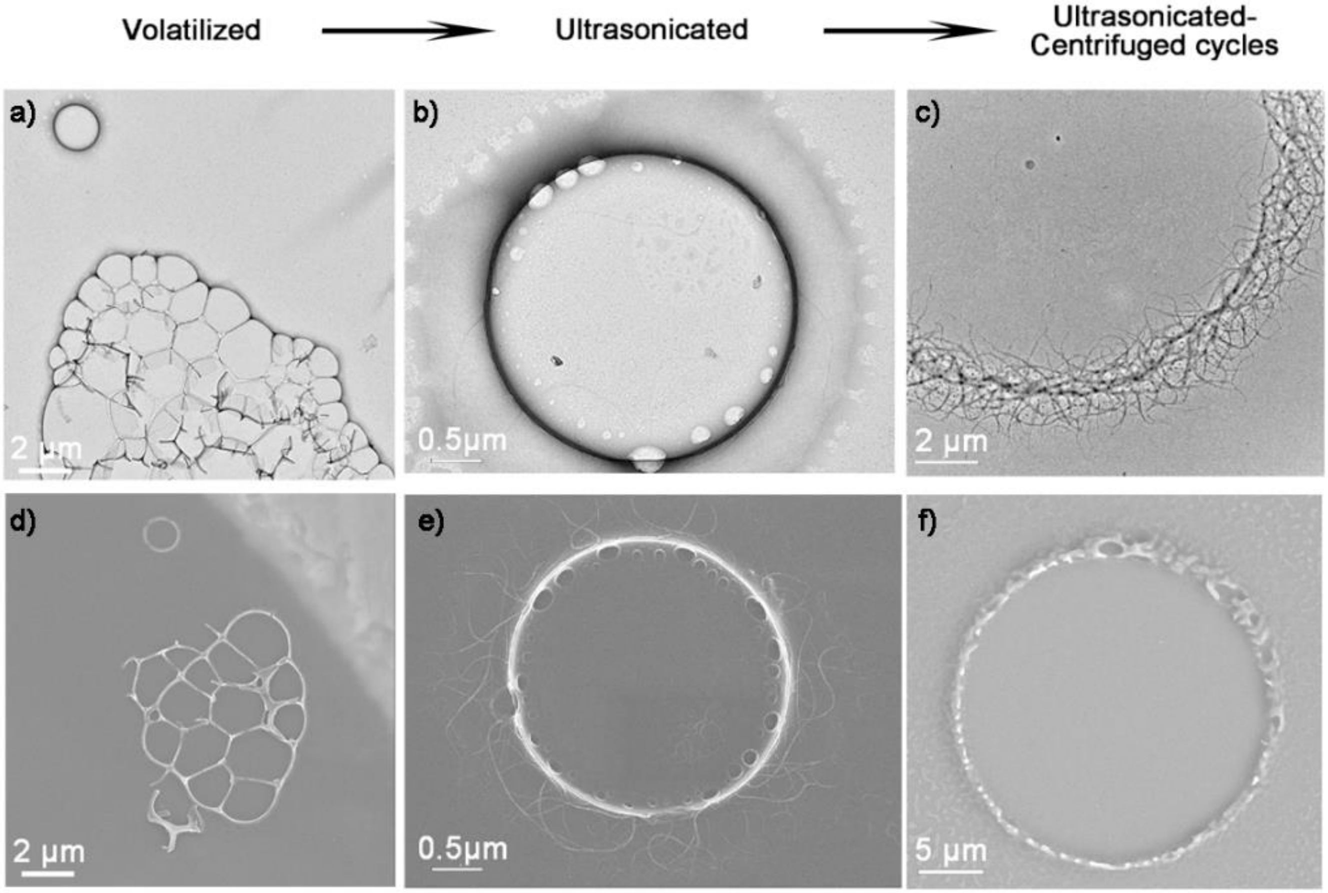

Supplementary Fig. 44. The TEM (a-c) and SEM (d-f) images of molecular cage 4*P*-HTMC in different morphology of tissue-like assemble structures (a, d), *flagellatas*-shaped like vesicles (b, e), and micro-scaled nanofiber rings (c. f).

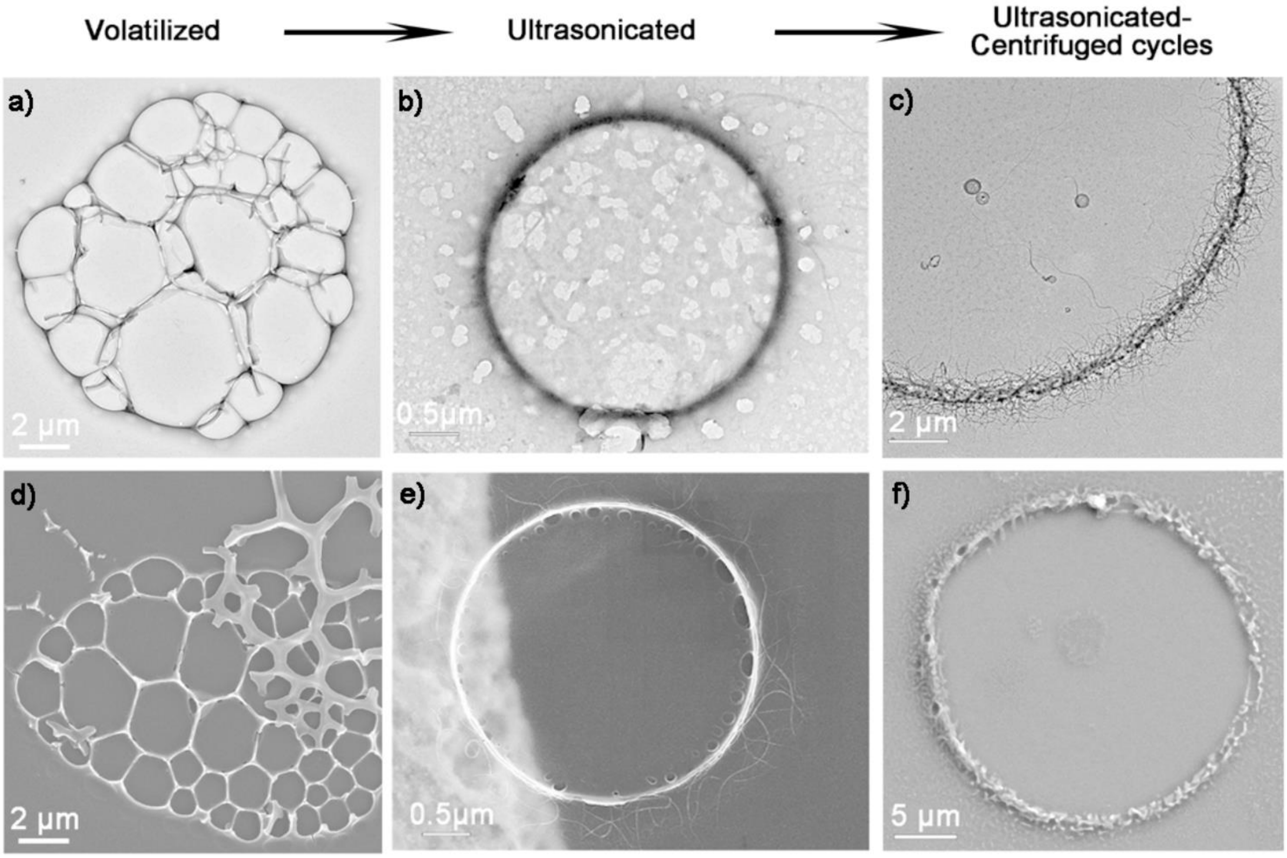

Supplementary Fig. 45. The TEM (a-c) and SEM (d-f) images of molecular cage 4*M*-HTMC in different morphology of tissue-like assemble structures (a, d), *flagellatas*-shaped like vesicles (b, e), and micro-scaled nanofiber rings (c. f).

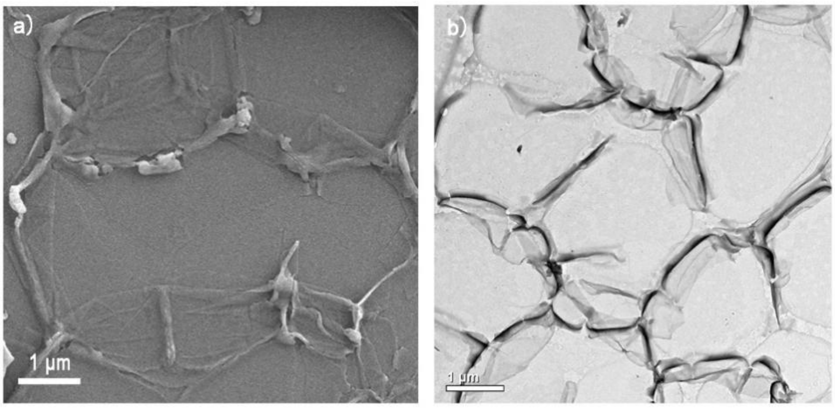

Supplementary Fig. 46. The SEM images (a) and TEM images (b) of molecular cage 4*P*-HTMC assemble into micro-scaled networks.

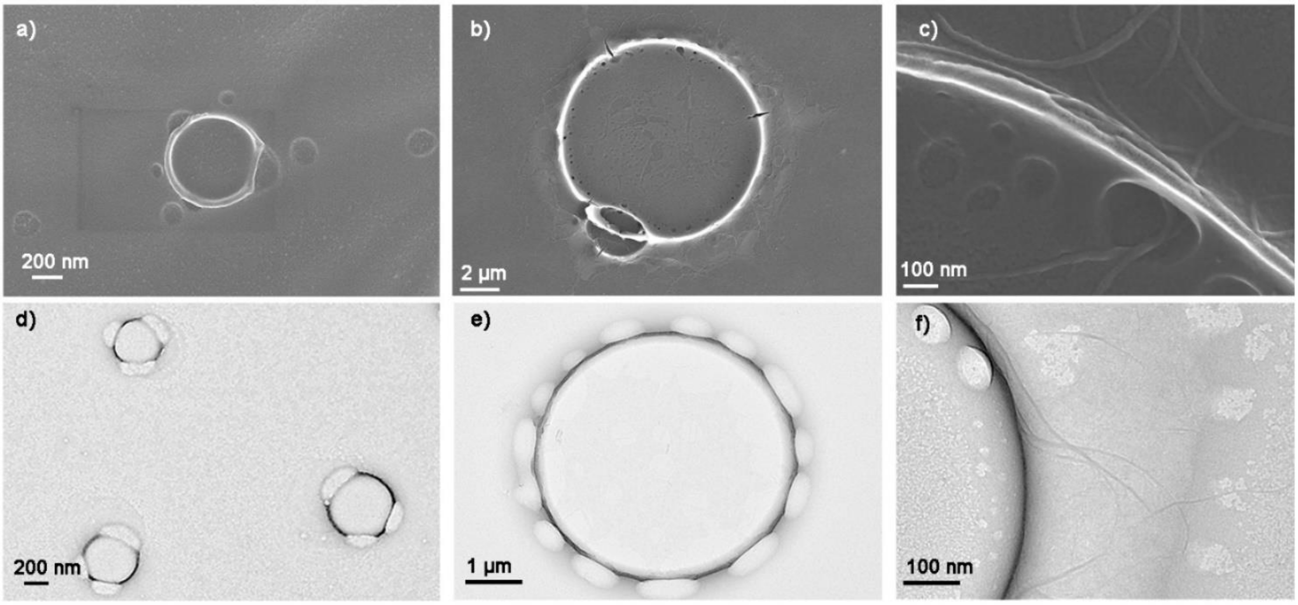

Supplementary Fig. 47. The SEM (a-c) images and TEM (d-f) images of molecular cage 4*P*-HTMC formed micro-vesicles in merging process and the nanofibers formed form the membrane.

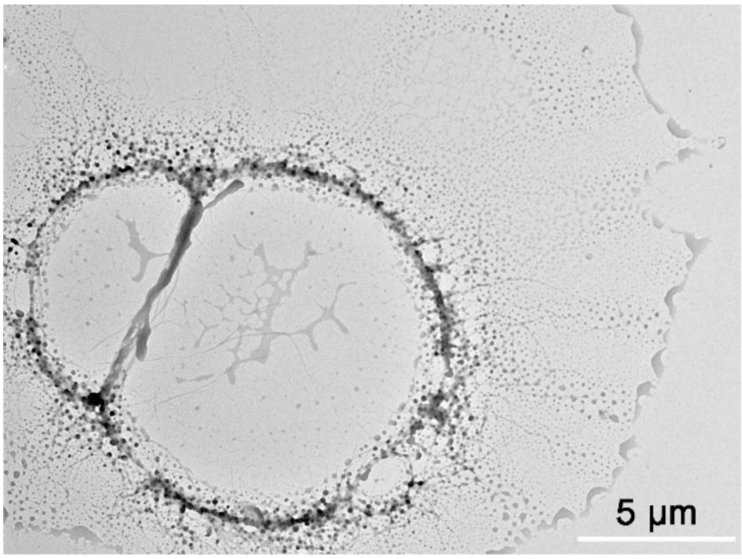

Supplementary Fig. 48. The TEM images of molecular cage 4*P*-HTMC formed huge ring structure in merging process.

11.PXRD

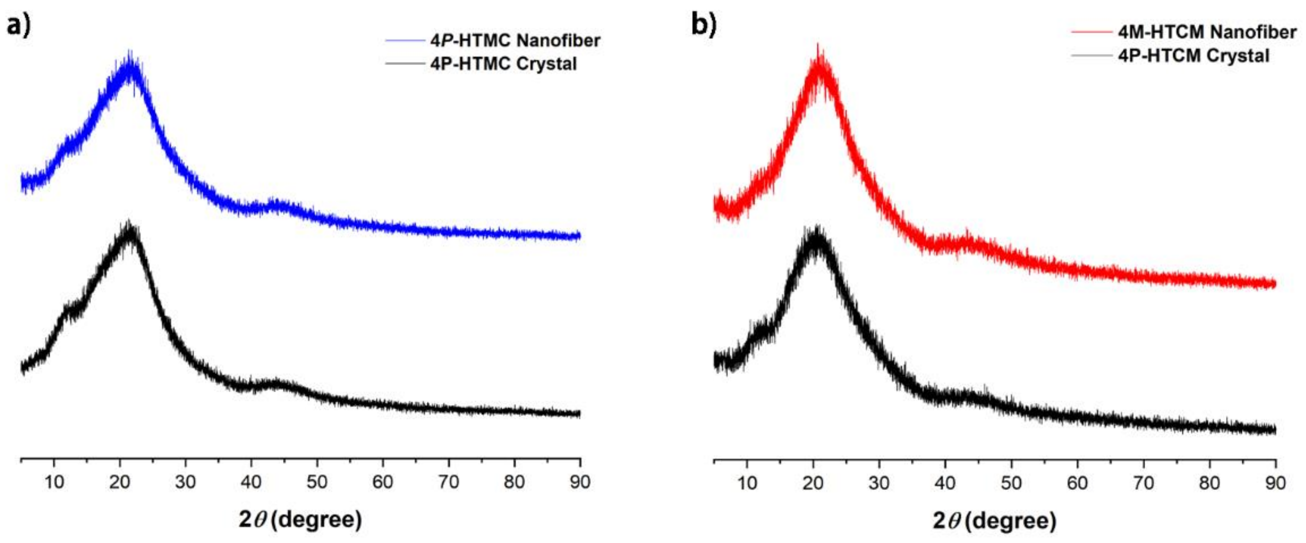

Supplementary Fig. 49. The PXRD patterns of a) molecular cage 4*P*-HTMC nanofiber (blue) and crystal solid (black), and b) 4*M*-HTMC nanofiber (red) and crystal solid (black).

References

1) Naseer, M. M.; Wang, D.-X.; Zhao, L.; Huang, Z.-T.; Wang, M.-X. Synthesis and Functionalization of Heteroatom-Bridged Bicyclocalixaromatics, Large Molecular Triangular Prisms with Electron-Rich and -Deficient Aromatic Interiors. *J. Org. Chem*. **2011**, 76, 1804–1813.
